# Binding of exogenous cyanide reveals new active-site states in [FeFe] hydrogenases†

**DOI:** 10.1039/d2sc06098a

**Published:** 2023-02-08

**Authors:** Maria Alessandra Martini, Konstantin Bikbaev, Yunjie Pang, Christian Lorent, Charlotte Wiemann, Nina Breuer, Ingo Zebger, Serena DeBeer, Ingrid Span, Ragnar Bjornsson, James A. Birrell, Patricia Rodríguez-Maciá

**Affiliations:** a Department of Inorganic Spectroscopy, Max Planck Institute for Chemical Energy Conversion Stiftstraße 34-36 45470 Mülheim an der Ruhr Germany maria.martini@cec.mpg.de; b Department of Chemistry and Pharmacy, Friedrich Alexander University Erlangen-Nürnberg Bioinorganic Chemistry Erlangen Germany; c College of Chemistry, Beijing Normal University 100875 Beijing China; d Institut für Chemie, Technische Universität Berlin Straße des 17. Juni 135 10623 Berlin Germany; e Ruanda-Zentrum und Büro für Afrika-Kooperationen, Universität Koblenz-Landau, Universitätsstraße 1 56070 Koblenz Germany; f Univ. Grenoble Alpes, CNRS, CEA, IRIG, Laboratoire de Chimie et Biologie des Métaux 17 Rue des Martyrs F-38054 Grenoble Cedex France; g School of Life Sciences, University of Essex Colchester CO4 3SQ UK james.birrell@essex.ac.uk; h Department of Chemistry, Inorganic Chemistry Laboratory, University of Oxford South Parks Road Oxford OX1 3QR UK patricia.rodriguezmacia@chem.ox.ac.uk

## Abstract

[FeFe] hydrogenases are highly efficient metalloenyzmes for hydrogen conversion. Their active site cofactor (the H-cluster) is composed of a canonical [4Fe-4S] cluster ([4Fe-4S]_H_) linked to a unique organometallic di-iron subcluster ([2Fe]_H_). In [2Fe]_H_ the two Fe ions are coordinated by a bridging 2-azapropane-1,3-dithiolate (ADT) ligand, three CO and two CN^−^ ligands, leaving an open coordination site on one Fe where substrates (H_2_ and H^+^) as well as inhibitors (*e.g.* O_2_, CO, H_2_S) may bind. Here, we investigate two new active site states that accumulate in [FeFe] hydrogenase variants where the cysteine (Cys) in the proton transfer pathway is mutated to alanine (Ala). Our experimental data, including atomic resolution crystal structures and supported by calculations, suggest that in these two states a third CN^−^ ligand is bound to the apical position of [2Fe]_H_. These states can be generated both by “cannibalization” of CN^−^ from damaged [2Fe]_H_ subclusters as well as by addition of exogenous CN^−^. This is the first detailed spectroscopic and computational characterisation of the interaction of exogenous CN^−^ with [FeFe] hydrogenases. Similar CN^−^-bound states can also be generated in wild-type hydrogenases, but do not form as readily as with the Cys to Ala variants. These results highlight how the interaction between the first amino acid in the proton transfer pathway and the active site tunes ligand binding to the open coordination site and affects the electronic structure of the H-cluster.

## Abstract 2

Binding of exogenous cyanide to the active site H-cluster in [FeFe] hydrogenase stabilizes new active-site states with an overoxidized diiron cofactor showing how σ-donor/π-acceptor interactions influence the electronic structure of metallocofactors.

## Graphical abstract

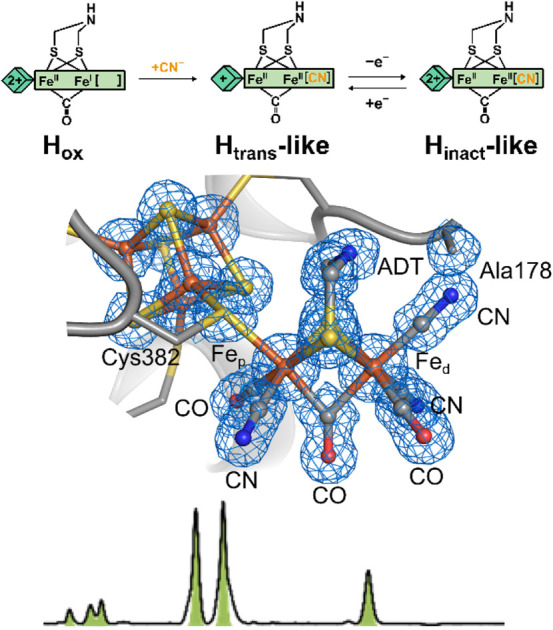

## Introduction

**Fig. 1:**
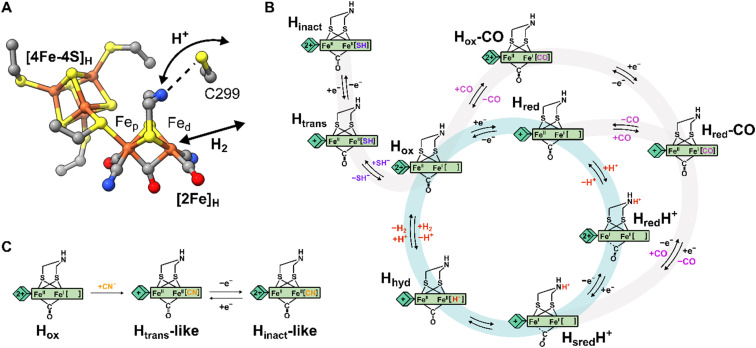
Structure of the H-cluster and proposed catalytic cycle. (A) Structure of the [2Fe]_H_ and [4Fe-4S]_H_ subclusters from *Clostridium pasteurianum* HydA1 (*Cp*HydA1, PDB: 4XDC)^1^ with the Cys in the proton transfer pathway also shown (C299 in *Cp*HydA1, C178 in *Dd*HydAB, C169 in *Cr*HydA1). (B) Proposed catalytic cycle also showing the pathways of reversible inactivation by H_2_S and CO, respectively. The pathway for H_inact_ formation is only applicable to *Dd*HydAB as the H_trans_ state has not been identified for other [FeFe] hydrogenases. A different catalytic cycle has been proposed by some authors.^6^ (C) Schematic showing the proposed chemical structure of the H_trans_-like and H_inact_-like states identified in this study.

Hydrogenases are the most powerful natural catalyst for the production and utilization of molecular hydrogen.^4,5^ Depending on the metal content of the cofactor at their active site, hydrogenases are classified as [FeFe], [NiFe] or [Fe] hydrogenases.^7^ For the [FeFe] type, the cofactor at the active site is called the H-cluster and consists of a canonical [4Fe-4S] cluster ([4Fe-4S]_H_) linked through a cysteine thiolate to a unique organometallic diiron cluster ([2Fe]_H_) (Fig. 1A).^8,9^ In [2Fe]_H_, the two irons (distinguished as proximal, Fe_p_, and distal, Fe_d_, depending on their relative distance from [4Fe-4S]_H_) are bridged by a 2-azapropane-1,3-dithiolate (ADT) ligand and a CO ligand, while additional CO and CN^−^ ligands are terminally bound to each Fe. The open coordination site at the apical position on Fe_d_ is where activation/formation of H_2_ occurs, but also where inhibitors including CO,^10^ H_2_S^2,11^ and O_2_ ^12^ bind. Binding of O_2_ generally leads to the destruction of the H-cluster, making [FeFe] hydrogenases highly oxygen-sensitive.^12–15^

Several states of the H-cluster differing in electron and proton distribution at the two subclusters and ligand binding to the open coordination site have been identified. However, the precise structure and the involvement of some of these states in the catalytic cycle (Fig. 1B) of [FeFe] hydrogenase is still a matter of debate.^6,16^ In [2Fe]_H_ the strong-field CO and CN^−^ ligands stabilize low-spin and low-oxidation states for the two Fe ions, which cycle between Fe(ii) and Fe(i) during catalysis. For instance, the active oxidized state H_ox_ has mixed valence Fe_p_(ii)Fe_d_(i) in [2Fe]_H_.^17^ The one-electron reduced state H_red_ retains the Fe_p_(ii)Fe_d_(i) valence in [2Fe]_H_ but has a reduced [4Fe-4S]_H_. The reduced states H_red_H^+^ and H_sred_H^+^ are thought to have an Fe_p_(i)Fe_d_(i) configuration at [2Fe]_H_ that is favored by concomitant protonation of the ADT ligand.^18–20^ The crucial two-electron reduced H_hyd_ state contains a terminal hydride on Fe_d_ and an overoxidized [2Fe]_H_ with a formal Fe_p_(ii)Fe_d_(ii) configuration.^21–25^ Recently, states with a terminal hydride on Fe_d_ differing in the redox state of [4Fe-4S]_H_ have been identified.^26^

A similar overoxidized [2Fe]_H_ can be found in two inactive states called H_trans_ and H_inact_.^11,27–30^ These states form upon reversible inactivation of [FeFe] hydrogenases by sulfide, which binds to the H-cluster under oxidizing conditions in some enzymes,^2,11^ or by binding of a nearby cysteine thiolate in others.^31–33^ In H_inact_, an RS^−^ ligand (where R can be H or the rest of the cysteine amino acid) is thus bound in the apical position to Fe_d_, [2Fe]_H_ is in the overoxidized state and the [4Fe-4S]_H_ subcluster is oxidized ([4Fe-4S]_H_^2+^–[Fe_p_(ii)Fe_d_(ii)-SR]_H_). Notably, the H_inact_ state is stable under air as the RS^−^ ligand prevents O_2_ from binding to Fe_d_. Reversible one-electron reduction of H_inact_, in which SH^−^ is bound, yields the H_trans_ state, which has a reduced [4Fe-4S]_H_^+^ ([4Fe-4S]_H_^+^–[Fe_p_(ii)Fe_d_(ii)-SH]_H_). Conversion of H_trans_ to the active hydrogenase appears to require an additional reduction step.^29^ However, the exact mechanism of conversion is not clear, but several theories have been proposed.^2,11,34^ So far, H_trans_ has not been identified in enzymes that bind a cysteine thiolate in H_inact_.^33^

Efficient exchange of protons between the solvent and the active site is crucial during H_2_ conversion and is facilitated by a proton channel (also called the proton transfer pathway, PTP). This pathway is formed by a series of largely conserved (at least in prototypical hydrogenases)^5^ amino acids and water molecules that form a network of hydrogen bonds connecting the protein surface with the H-cluster.^35–37^ Site-directed mutagenesis of amino acids along the proton transfer pathway can impair or even completely abolish catalytic activity, as a consequence of the slower proton exchange with the H-cluster.^36,38–40^ Additionally, it was observed that some H-cluster states accumulate differently in wild-type enzymes and in variants with deficient proton transfer. Closest to the H-cluster is a cysteine residue (C299 in *Clostridium pasteurianum* HydA1, C178 in *Desulfovibrio desulfuricans* HydAB, *Dd*HydAB, and C169 in *Chlamydomonas reinhardtii* HydA1) whose thiol is within hydrogen-bond distance of the bridgehead amine of the ADT ligand (Fig. 1A). When this Cys was mutated to alanine (Ala) or serine (Ser) in *Chlamydomonas reinhardtii* HydA1 (*Cr*HydA1 C169A and C169S) and to Ser in *Clostridium pasteurianum* HydA1 (*Cp*HydA1 C299S), these hydrogenases formed readily the H_hyd_ state.^21–23,39,41,42^ In addition, the *Cr*HydA1 C169S variant was shown, using electron paramagnetic resonance (EPR) spectroscopy, to accumulate a state similar to H_trans_, but the precise nature of this state remains unknown.^21,22,43^ Finally, the C169A variant of *Cr*HydA1 has been reported to react with oxygen to form an H_ox_–O_2_ state (so far observed only in this particular mutant), which has been suggested to have a superoxide bound to Fe_d_ and an oxidized [4Fe-4S] cluster, yielding a [4Fe-4S]_H_^2+^–[Fe_p_(i)Fe_d_(iii)-O_2_^−^]_H_ electronic configuration.^44^ However, the infrared (IR) spectrum of this state is very similar to the H_inact_ state and so a formal Fe_p_(ii)Fe_d_(ii) valence would seem more likely.

In this study, we investigated the effects of replacing the Cys in the proton transfer pathway with alanine in the hydrogenases *Dd*HydAB and *Cr*HydA1. *Dd*HydAB is an exceptionally active bidirectional hydrogenase that contains two additional [4Fe-4S] clusters (F-clusters) for electron transfer between the H-cluster and the protein surface. The mutation of amino acids along the proton transfer pathway of *Dd*HydAB has not been investigated before. The C169A variant of *Cr*HydA1 has already been studied in particular in relation to the H_hyd_ and H_ox_–O_2_ states,^23,41,44^ but here we report two new active site states in *Cr*HydA1 C169A never identified before. In both *Dd*HydAB C178A and *Cr*HydA1 C169A, we observed formation of unprecedented H-cluster states similar to H_trans_ and H_inact_. By combining their spectroscopic and structural characterization, we demonstrated that these H_trans_-like and H_inact_-like states form upon binding of CN^−^ to the H-cluster (Fig. 1C). These CN^−^-bound states form also in wild-type (WT) hydrogenases, but are stabilized in the Cys to Ala mutants. This study highlights how the interaction between the Cys in the proton transfer pathway and the H-cluster (specifically the bridgehead amine in [2Fe]_H_) tunes the electronic structure of the H-cluster and regulates ligand binding to the apical position of Fe_d_.

## Results

### *Dd*HydAB C178A is isolated in an H_trans_-like state

**Fig. 2:**
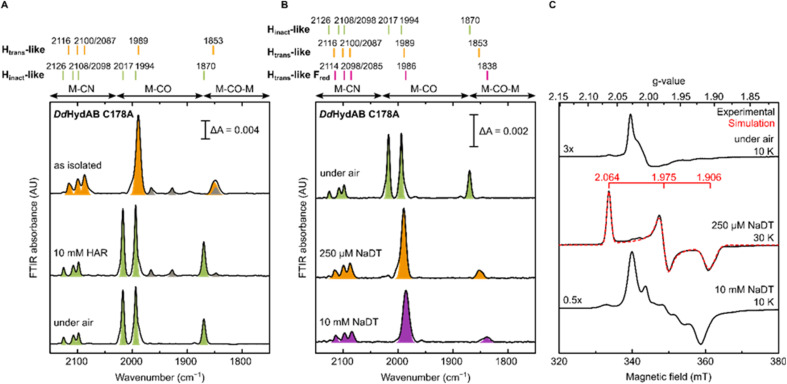
IR and EPR spectra of *Dd*HydAB C178A exhibit new H_trans_-like and H_inact_-like states. (A) IR spectra of freshly maturated *Dd*HydAB C178A (in 25 mM Tris pH 8.0, 25 mM KCl): as isolated; oxidized under anaerobic conditions (with 10 mM HAR); oxidized under air. Bands from the H_trans_-like and H_inact_-like states are colored in orange and green, respectively. (B and C) Samples of *Dd*HydAB C178A were prepared under different conditions. One aliquot was used for room temperature IR measurements (B), while the remaining sample was used for CW X-band EPR measurements (C). In (A and B), bands are color coded as follows: green for H_inact_-like state, orange for H_trans_-like state, purple for H_trans_-like F_red_ state. The gray bands correspond to traces of an unidentified state that potentially lacks the third CN^−^ ligand (which protects the H-cluster from O_2_ attack), as this state disappears after exposure to air. In (C), experimental spectra are in black, overlaid in one case with spectral simulations (dashed red line). EPR experimental conditions: microwave frequency = 9.64 GHz; microwave power = 1 mW for the first two conditions (under air, 250 μM NaDT), 0.1 mW for the bottom one (10 mM NaDT); temperature is specified in the figure.

The *Dd*HydAB C178A mutant was recombinantly expressed in *E. coli* as an “apo”-hydrogenase (*i.e.* containing the [4Fe-4S]_H_ subcluster and all the accessory F-clusters but lacking [2Fe]_H_) and artificially maturated *in vitro*, as routinely performed with the WT enzyme.^45^ The WT *Dd*HydAB is commonly isolated after maturation (under 2% H_2_ and 98% N_2_) as a mixture of states, mainly H_ox_, H_ox_–CO, and H_red_H^+^.^45^ As shown in the IR spectra in Fig. 2A, after artificial maturation the C178A mutant was, surprisingly, isolated in an almost pure unprecedented state that greatly differs from the states normally observed in freshly maturated WT *Dd*HydAB (Table S1†). In particular, the spectrum of the as isolated C178A mutant exhibits a broad absorption at 1853 cm^−1^ attributed to the bridging CO ligand, a single broad band at 1989 cm^−1^ attributed to the terminal CO ligands (potentially resulting from the two overlapping CO bands, exhibiting a shoulder at ∼2002 cm^−1^), and three bands at 2116, 2100 and 2087 cm^−1^ attributed to CN^−^ ligands (instead of the expected two bands). Relative to the IR bands observed for the H_ox_ state in WT *Dd*HydAB, the IR bands of the C178A variant are shifted to higher energies (blue-shifted) suggesting decreased electron density on [2Fe]_H_. The frequencies of the IR bands are very similar to those of the H_trans_ state in WT *Dd*HydAB (Table S1†),^2,29^ therefore, we hypothesized that this new state could be an H_trans_-like state (*i.e.* with the same electronic configuration [4Fe-4S]_H_^+^–[Fe_p_(ii)Fe_d_(ii)]_H_). An H_inact_-like state ([4Fe-4S]_H_^2+^–[Fe_p_(ii)Fe_d_(ii)]_H_) was formed both by oxidation of the as-isolated C178A variant under anaerobic conditions (using hexaammineruthenium(iii) chloride, HAR) or by exposure to atmospheric oxygen (Fig. 2A, green bands), yielding identical IR spectra in each case. Exposure of the as-isolated sample to air did not result in any significant decrease in IR signal intensity, suggesting that all active site species present in the H_trans_-like state transform into an H_inact_-like state. Notably, this H_inact_-like state is another example of an air-stable state in [FeFe] hydrogenases. Therefore, in addition to having the same electronic configuration of H_trans_ and H_inact_, respectively, we hypothesized that the new states might have an additional ligand (likely different from SH^−^) bound at the open coordination site.

To confirm our assignment of H_trans_-like and H_inact_-like states, we measured EPR spectra of *Dd*HydAB C178A poised in different states as confirmed by IR spectroscopy (Fig. 2C). The H_inact_-like state is EPR silent, like the H_inact_ state in WT *Dd*HydAB.^30^ Only a signal from a [3Fe-4S] cluster could be detected, probably due to oxidative damage to the F-cluster located in vicinity of the protein surface. Addition of one equivalent of reducing agent (sodium dithionite, NaDT) to the *Dd*HydAB C178A sample, which had partially converted to the H_inact_-like state during storage (Fig. S1A†) reverts it to the H_trans_-like state (Fig. 2B). The H_trans_-like state exhibits a rhombic EPR signal (*g* = 2.06, 1.98, 1.91) similar to the one of H_trans_ in WT *Dd*HydAB (Fig. S2†),^30^ confirming our initial assignment of *Dd*HydAB C178A being isolated in an H_trans_-like state. Addition of excess NaDT results in only minor shifts to lower energies (red-shift) of all IR bands and gives rise to a complex EPR interaction spectrum. We interpreted this behaviour with the H-cluster remaining in an EPR-active H_trans_-like state while the accessory F-clusters are being reduced to EPR-active states by the excess of NaDT, resulting in strong dipolar spin-coupling (H_trans_-like F_red_ state) as observed previously for the WT enzyme in the H_ox_ or H_ox_–CO state with reduced F-clusters.^46^ Samples in the as-isolated state and purged with CO showed no change to the IR spectrum (Fig. S1B†) indicating that this state was unable to bind CO, most likely due to an already occupied coordination site.

### Evidence for an additional CN^−^ ligand at the H-cluster in *Dd*HydAB C178A

**Fig. 3:**
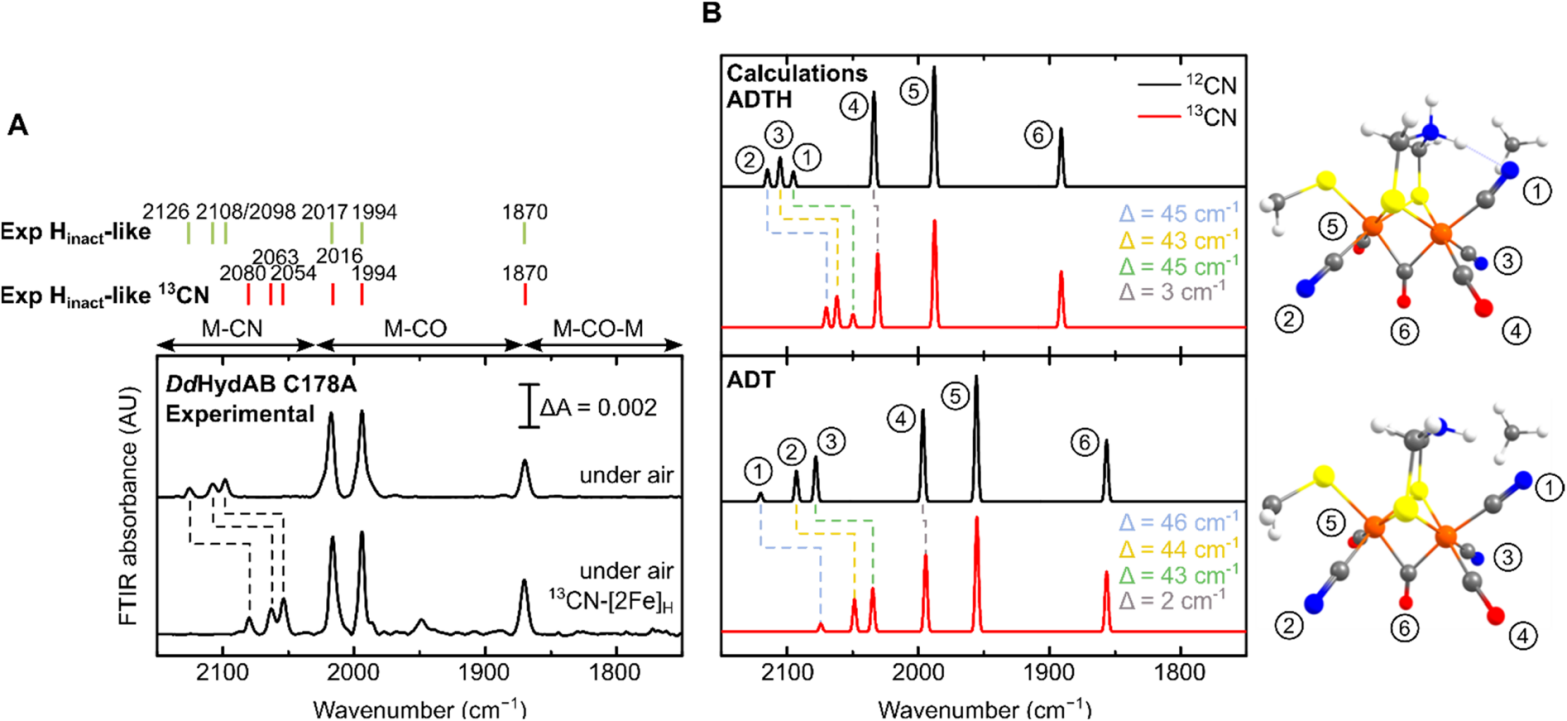
(A) Experimental IR spectra of *Dd*HydAB C178A maturated with natural abundance (top) and ^13^CN^−^-labeled [2Fe]_H_ precursor (bottom), both exposed to air to form the H_inact_-like state. The small additional feature in the ^13^CN spectrum may represent a small amount of an unknown degradation product. (B) Calculated IR spectra of the H-cluster model with either a singly protonated (ADT) or doubly protonated (ADTH) ADT ligand. Insets are [2Fe]_H_ structures with chemical groups associated with modes labelled. A scaling factor of 0.964 was used.

For all the states we could observe in *Dd*HydAB C178A (H_trans_-like, H_inact_-like and H_trans_-like F_red_), the IR spectra always exhibit three bands in the CN^−^ region. To test if all the bands derive from CN^−^ vibrations associated with the H-cluster, we performed the artificial maturation of the C178A mutant with a precursor of the [2Fe]_H_ cluster with both CN^−^ ligands labelled with ^13^C. As shown in Fig. 3A, we observed an isotope shift (46–44 cm^−1^) of all three CN^−^ absorptions in the IR spectrum of the H_inact_-like state. We could interpret these results in three ways: (i) three CN^−^ ligands are present at the H-cluster; (ii) two CN^−^ vibrations couple in an unusual way giving rise to three IR bands; (iii) spectra represent two very similar states with one strongly overlapping CN^−^ band, while the other CN^−^ band in each state is distinct. The second hypothesis is not likely as the CN^−^ ligands are on different Fe ions and such a structure is unlikely to give significant quadratic coupling.^29,47^ Previous isotope editing experiments on the CO ligands in WT enzyme showed very little perturbation in the vibrational frequency of the pCO ligand (the terminal CO on Fe_p_), when dCO (the terminal CO on Fe_d_) or μCO (the bridging CO) were exchanged with ^13^CO.^29,47^

The isotope shift could be reproduced by quantum mechanics/molecular mechanics (QM/MM) calculations of an H-cluster model (Fe(ii)Fe(ii) redox state) of the C178A variant of *Dd*HydAB, with CN^−^ as the exogenous ligand on Fe_d_, as shown in Fig. 3B. Two protonation states of the amine in the ADT ligand were calculated: singly protonated (ADT) and doubly protonated (ADTH). The experimental ^13^CN isotope shifts of 43–46 cm^−1^ could be satisfactorily reproduced with both models: 43–46 cm^−1^ (ADT) and 43–45 cm^−1^ (ADTH). The absolute experimental frequencies are reasonably well reproduced by scaled harmonic frequencies, though with some differences between ADT and ADTH models. The terminal CO modes were somewhat better predicted by the ADTH model while the CN^−^ modes and bridging CO modes were better predicted with the ADT model. We note that the CO frequencies are quite dependent on the quality of the model, density functional and scaling factor while the CN^−^ frequencies are less so (Fig. S3 and Materials and methods in the ESI†). The calculated relative intensities of the three CN^−^ modes, however, differ more strongly between models. The ADT model predicts an increase in CN^−^ mode intensity with decreasing energy, consistent with the experimental intensities, while the ADTH model does not. The reason is that the order of the assigned CN^−^ modes differs between ADT/ADTH models; the exogenous CN^−^ mode is the highest-energy CN^−^ mode for the ADT model but it is the lowest for the ADTH. These differences can be explained by a stronger exogenous CN^−^-binding in the ADTH model (aided by stronger H-bonding to the doubly protonated amine). Other conformers of the ADT and ADTH models were explored (Fig. S4†) but were found to be energetically unfavorable. Overall the calculations suggest the H_inact_-like state as best described by an [Fe_p_(ii)Fe_d_(ii)]_H_ model featuring an exogenous CN^−^ ligand in the apical position with a singly protonated bridgehead amine of the ADT ligand.

**Fig. 4:**
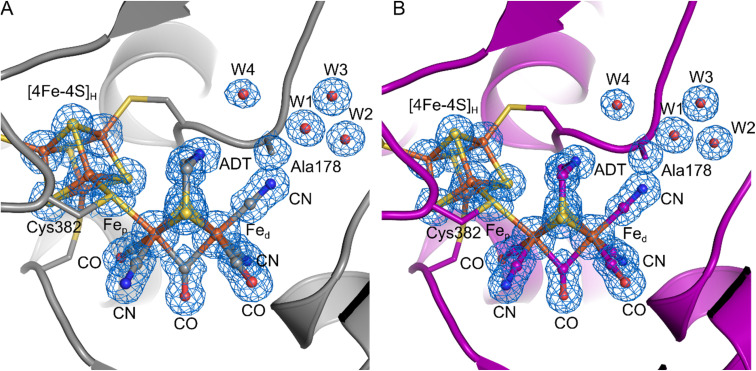
Crystal structures of the *Dd*HydAB C178A mutant in two different states. (A) *Dd*HydAB in the H_inact_-like state (PDB ID 8BJ7) is shown as cartoon and colored in gray. (B) *Dd*HydAB in the H_trans_-like state (PDB ID 8BJ8) is shown as cartoon and colored in magenta. Close-up view of the active site showing the H-cluster, the Cys ligating the cofactor, the side chain of Ala178 and the well-defined water molecules with a distance <4.0 Å from Ala178. The protein backbone is shown as cartoon, the amino acid side chains and the H-cluster including the bound CN^−^ are shown as stick model, and water molecules are shown as spheres. The cofactors and amino acid side chains are colored according to the element-specific color code. A 2Fo–Fc electron density map (blue mesh, contoured at 1.0*σ*) is shown for the H-cluster including the CN^−^ ligand, the side chain of Ala178, and the water molecules.

To further investigate the properties of the H-cluster in the C178A variant of *Dd*HydAB, we solved crystal structures of the enzyme in the H_inact_-like and the H_trans_-like states using X-ray crystallography (Fig. 4 and S5–S9†). After exposure to air to form the H_inact_-like state, the protein was crystallized under aerobic conditions (Fig. 4A and S7†) as performed previously for the SH-bound H_inact_ state in the WT enzyme.^11^ IR and resonance Raman (RR) measurements on crystals^48^ prepared under the same conditions confirmed that the enzyme was indeed in the H_inact_-like state (Fig. S5†). However, small shifts in the band positions compared with solution measurements were observed and are likely related to temperature-dependent changes or crystal packing effects. Such effects have been observed previously for [FeFe]^11,49^ and [NiFe] hydrogenases.^48,50,51^ We also solved a structure of the H_trans_-like state of the enzyme from crystals grown under anaerobic conditions (2% H_2_, 98% N_2_) (Fig. 4B). *Dd*HydAB C178A crystallized in an orthorhombic space group *P*2_1_2_1_2_1_ as observed for the previously reported WT *Dd*HydAB in the H_inact_ state.^11^ The crystals diffracted up to 1.0 Å and the structures were solved at high resolution, 1.04 Å for the H_inact_-like state and 1.01 Å for the H_trans_-like state. The overall structure of the C178A variant in the H_inact_-like state and the WT enzyme in the H_inact_ state is virtually identical with an RMSD of 0.237 Å (calculated for all Cα atoms of all residues, Table S2) (Fig. S7 and S8†). The structure at atomic resolution clearly shows a diatomic ligand in the apical position of Fe_d_ and the Ala residue that replaced the Cys at position 178. Moreover, we identified two additional well-defined water molecules appearing near the Ala178. Interestingly, in the crystal structure of the C299A variant from *Cp*HydA1 reported by Duan *et al.*,^36^ the space of the missing thiol group was replaced by an additional H_2_O molecule (Wat962), which was located at 3.4–3.7 Å from the NH group of the ADT ligand and at 3.6–3.7 Å from the Wat826 molecule of the PTP.^36^ This new water molecule was hypothesised to rescue proton transfer activity in the absence of the thiol; however, the authors could not measure any significant catalytic activity.

The overall architecture of the H_inact_-like and the H_trans_-like states are also virtually identical with an RMSD of 0.053 Å (calculated for all Cα atoms of all residues, Table S2) (Fig. S8†). While for the structure of WT *Dd*HydAB in the H_inact_ state a reduced occupancy of the [2Fe]_H_ subcluster led to better agreement between modelled and experimental data,^11^ here we observed no negative difference density when refining the structural models with an occupancy of 100% for the [2Fe]_H_ subcluster. This could be evidence for a better incorporation of the [2Fe]_H_ subcluster during artificial maturation or higher stability of the H-cluster during crystallization in the C178A mutant compared to the WT protein.

The crystal structure clearly shows a well-defined, relatively symmetric bridging CO (Fig. S9†), with roughly equal Fe_p_—C_b_ and Fe_d_—C_b_ bond distances. A similar observation was made for the H_inact_ state in wild type *Dd*HydAB,^11^ whereas other [FeFe] hydrogenase structures show slight lengthening of the Fe_p_—C_b_ bond and shortening of the Fe_d_—C_b_ bond.^8,9,52^ The differences here are attributed to the oxidation states and coordination environment of Fe_p_ and Fe_d_. In our structure with cyanide bound and the previously published H_inact_ state^11^ both Fe ions were Fe(ii) and hexacoordinate. Meanwhile for structures obtained of the active enzyme,^8,9,52^ the Fe ions are more reduced (for H_ox_ Fe_d_ is reduced to Fe(i) and for H_red_H^+^ both Fe ions are reduced to Fe(i)) and Fe_d_ is pentacoordinate. These effects lead to shortening of the Fe_d_—C_b_ bond giving a semi-bridging CO.

On the basis of these results and the fact that the IR spectra of all H_inact_-like and H_trans_-like states in *Dd*HydAB C178A exhibit three CN^−^ bands, we suggest that in these states a third CN^−^ is present at the H-cluster, bound at the apical position on Fe_d_. Therefore, in the structure we modelled a CN^−^ ligand coordinated to the distal iron ion through its carbon atom, with a Fe–C distance of 1.90 Å. To confirm the assignment of the ligands, we calculated an omit map in the absence of the [2Fe]_H_ subsite and the additional ligand at the apical position on Fe_d_. The omit map (Fig. S9†) supports the positioning of the atoms in the electron density. In addition, we are able to distinguish between the N and O atoms of the terminal and bridging ligands when increasing the contouring level to *σ* = 2.8 Å (Fig. S9C†). This result suggests that the exogenous CN^−^ ligand remains in the apical position, which agrees with the QM/MM calculations as well as the illumination experiments on *Cr*HydA1 C169A (see below).

### A similar H_trans_-like state in *Cr*HydA1 C169A

**Fig. 5:**
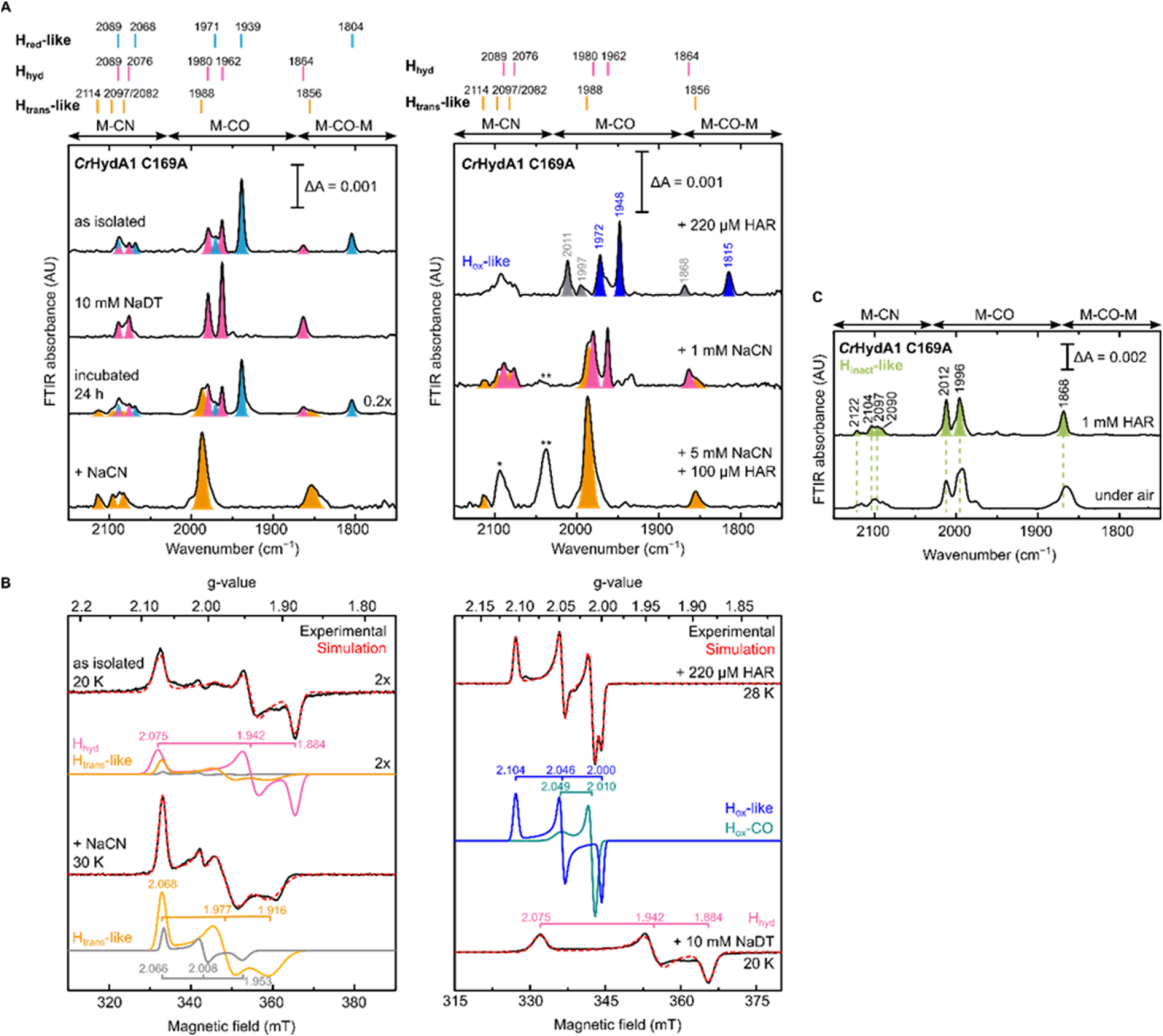
IR and EPR spectra suggest formation of a CN^−^-dependent H_trans_-like state also in *Cr*HydA1 C169A. (A) Room temperature IR spectra of *Cr*HydA1 C169A under different conditions: as isolated; with NaDT; after 24 h incubation under 2% H_2_; after addition of 5 mM NaCN, 100 μM HAR and then buffer exchanged (“+NaCN”); oxidized with 220 μM (1.1 eq.) HAR; after addition of 1 mM NaCN; after addition of 5 mM NaCN and 100 μM (0.5 eq.) HAR. Bands are color-coded as follows: light blue for H_red_-like, pink for H_hyd_, orange for the H_trans_-like, and blue for the H_ox_-like state. Bands in gray correspond to traces of the H_inact_-like and H_ox_–CO states. For the H_ox_-like state the bands in the complex CN^−^ region could not be assigned. In the H_hyd_ state, CN^−^ binding is likely disfavored as a hydride is bound to Fe_d_. The single asterisk marks the band of HCN (2093 cm^−1^), while the double asterisk marks the band of [Fe(CN)_6_]^4−^ (2037 cm^−1^),^3^ which suggests partial cofactor degradation upon CN^−^ addition. Clean spectra for the H_trans_-like state where obtained after buffer-exchanging the protein to eliminate HCN, free CN^−^ and degradation products. (B) CW X-band EPR spectra for some of the conditions shown in (A). Experimental spectra are shown in black and are overlaid with spectral simulations (dashed red line) with component spectra underneath. The pink component corresponds to the H_hyd_ state. The orange component is likely the H_trans_-like state, while the gray component may represent an alternative, as yet unidentified, state. Presence of the H_trans_-like components in the EPR spectrum of as isolated enzyme suggests that a small amount of H_trans_-like state has already formed in freshly maturated *Cr*HydA1 C169A (the small shoulder at 1988 cm^−1^ in the IR spectrum of the same sample is also consistent with the presence of traces of the H_trans_-like state). The blue trace corresponds to the H_ox_-like state, while the dark cyan trace corresponds to the H_ox_–CO state. EPR experimental conditions: microwave frequency = 9.64 GHz; microwave power = 1 mW; temperature is specified in the figure. (C) IR spectra of the H_inact_-like states in *Cr*HydA1 C169A. From the H_trans_-like state, two similar but slightly different H_inact_-like states form by oxidation of the enzyme with HAR under anaerobic (top) or by oxidation by atmospheric oxygen (bottom).

To gain further understanding of these CN^−^ bound states and on the role of the Cys in the proton transfer pathway in their formation, we decided to re-investigate the C169A variant of *Cr*HydA1. As mentioned in the introduction, this particular mutation in *Cr*HydA1 has already been studied and showed accumulation of the H_hyd_ and H_ox_–O_2_ states.^23,41,44^ In contrast with what we observed with *Dd*HydAB C178A, and consistent with the previous reports on *Cr*HydA1 C169A, this mutant is isolated after artificial maturation without an additional CN^−^ ligand on the H-cluster. Indeed, *Cr*HydA1 C169A is initially isolated under 2% H_2_ in a mixture of the H_hyd_ state (which can be enriched upon addition of NaDT) and another state that has been previously assigned as H_ox_ on the basis of the position of the IR bands (Fig. 5A).^23,53^ However, the EPR spectrum of the as-isolated enzyme lacks the characteristic rhombic signal for the H_ox_ state and shows only the H_hyd_ state as major component (75%) (Fig. 5B). The EPR spectrum of the H_hyd_ state in *Cr*HydA1 C169A (*g* = 2.075, 1.942, 1.884) is nearly identical to the one reported for the same state in the C169S mutant (*g* = 2.07, 1.94, 1.88).^22^ Therefore, we suggest that the state originally labelled as H_ox_ may instead be an EPR silent state with a similar electronic structure to the H_red_ state (H_red_-like, light blue IR bands in Fig. 5A), which has a reduced [4Fe-4S]_H_ ([4Fe-4S]_H_^+^–[Fe_p_(ii)Fe_d_(i)]_H_). Oxidation of this state with one equivalent of oxidizing agent (HAR) under anaerobic conditions formed a new state with its most intense IR band at 1948 cm^−1^, with an EPR spectrum (*g* = 2.104, 2.046, 2.000) similar to that observed for the H_ox_ state in WT *Cr*HydA1 (dark blue bands in Fig. 5A). Interestingly, these H_red_-like and H_ox_-like states have similar FTIR spectra to those observed for the recently characterised H_ox_H and H′_red_H states,^53–55^ with bands shifted to higher energy compared with the H_red_ and H_ox_ states in WT *Cr*HydA1.

**Fig. 6:**
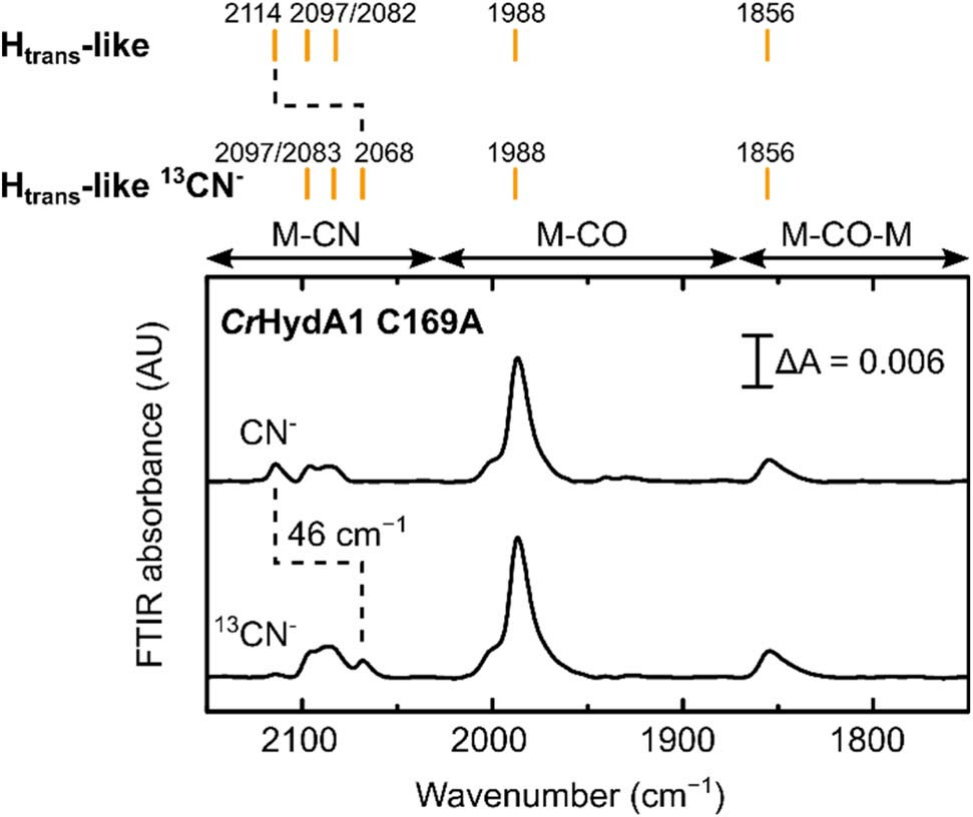
IR spectra of *Cr*HydA1 C169A prepared in the H_trans_-like state using natural abundance and ^13^C labelled CN^−^ exhibit an expected isotope shift of one of the CN^−^ bands. Small peaks in the region between 1930 and 1940 cm^−1^ are likely due to small contributions from states without CN^−^ bound.

Despite being initially isolated in states lacking an additional CN^−^ ligand (H_red_-like, H_hyd_), incubation of *Cr*HydA1 C169A (pH 8) in the glovebox (2% H_2_, 98% N_2_) for 24 h at room temperature led to the appearance in the IR spectrum of an H_trans_-like state similar to the one observed in *Dd*HydAB C178A, including a third CN^−^ band appearing at high energy (2114 cm^−1^, Fig. 5A). This H_trans_-like state could also be enriched upon addition of exogenous CN^−^ to freshly maturated enzyme (Fig. 5A), which is initially isolated as a mixture of H_red_-like and H_hyd_ (Fig. 5A). CN^−^ binding to the H_hyd_ state seems less favored, consistent with the presence of a terminal hydride bound to Fe_d_ in H_hyd_ (Fig. 5A). Therefore, complete conversion of the as-isolated enzyme to the H_trans_-like state required addition of half an equivalent of oxidizing agent (HAR) to first oxidize the H_hyd_ state (Fig. 5A). Addition of excess CN^−^ induces partial degradation of the H-cluster, as demonstrated by the appearance in the IR spectra of a broad band around 2037 cm^−1^, which suggests formation of [Fe(CN)_6_]^4−^ (Fig. 5A),^3^ as already reported for other Fe-containing metalloenzymes like CODH upon treatment with CN^−^.^56^ Therefore, after the formation of the H_trans_-like state, samples were buffer exchanged to remove degradation products as well as the excess of free CN^−^ to give cleaner IR spectra as in Fig. 5 and 6.

The EPR spectrum of the H_trans_-like state in *Cr*HydA1 C169A could be simulated with two components having similar rhombic signals (Fig. 5B). The first component (*g* = 2.068, 1.977, 1.916), accounting for *ca.* 87% of the signal, resembles the EPR spectrum of the H_trans_-like state in *Dd*HydAB C178A as well as a similar H_trans_-like state previously observed in *Cr*HydA1 C169S (*g* = 2.065, 1.969, 1.906), which was never assigned to a particular structure of the H-cluster.^21,22,43^ The second rhombic component (*g* = 2.066, 2.008, 1.935) could potentially relate to a different protein or H-cluster conformation. As observed for *Dd*HydAB C178A, oxidation of *Cr*HydA1 C169A in the H_trans_-like state yields an H_inact_-like state. However, two similar H_inact_-like states were formed depending on whether the oxidation was performed under anaerobic conditions by HAR or by atmospheric oxygen (Fig. 5B). A reason for this difference could be damage to the [4Fe-4S]_H_ during the different oxidative treatments. Notably, illumination of the air-oxidized *Cr*HydA1 C169A at cryogenic temperature (Fig. S10†) did not reveal any photosensitivity, confirming again the assignment of a terminally bound CN^−^ for the H_trans_-like and H_inact_-like states, since a CO species in the apical position would likely be photolyzed.^29,30^

Treatment of freshly-maturated *Cr*HydA1 C169A with ^13^CN^−^ yielded an H_trans_-like state which exhibited an isotope shift (46 cm^−1^) of one of the CN^−^ band in the IR spectrum (Fig. 6). This result unambiguously confirms that a third CN^−^ ligand is bound at the H-cluster in the H_trans_-like and H_inact_-like states. Additionally, this result allows us to assign the exogenous CN^−^ ligand on Fe_d_ to the band with the highest vibrational frequency (in samples prepared with ^12^CN^−^) among the three CN^−^ bands. In line with this observation, resonance Raman (RR) measurements on *Cr*HydA1 C169A in the H_inact_-like state (Fig. S11A†) revealed 1–3 cm^−1^ downshifts in the region characteristic for metal–ligand vibrations with contributions from the cyanide ligands (390–600 cm^−1^), when comparing samples prepared with ^12^CN^−^ and ^13^CN^−^. This is consistent with an expected lower vibrational frequency in the presence of a heavier atom. Calculated Raman spectra of the apical CN^−^-bound H-cluster models in a [Fe_p_(ii)Fe_d_(ii)]_H_ redox state (note: using the *Dd*HydAB QM/MM model) reproduce the experimental RR spectra and ^13^C isotope shifts fairly well (Fig. S11†). An observed mode at 603 cm^−1^ (experimental), assigned as a bridging CO bending mode, could only be reproduced (calculated 605 cm^−1^) with the ADT model, suggesting the ADT ligand to be singly protonated.

### Addition of CN^−^ to WT hydrogenases

**Fig. 7:**
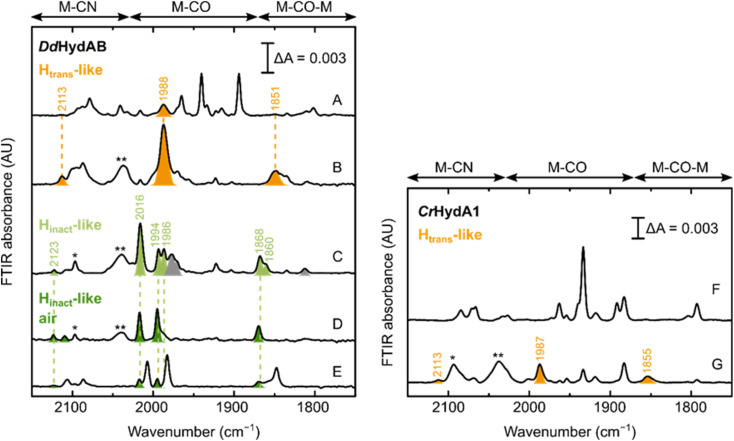
Formation of the H_trans_-like and H_inact_-like states in WT [FeFe] hydrogenase. IR spectra of WT *Dd*HydAB and WT *Cr*HydA1 under several conditions. Left panel – *Dd*HydAB: as isolated (A); with 10 mM NaCN (B); with 10 mM NaCN and 10 mM HAR (C); with 10 mM NaCN, 10 mM HAR and after exposure to air (D); after preparation of the H_inact_ state with Na_2_S as described in ref. 2. Right panel – *Cr*HydA1: as isolated (F) and with 10 mM NaCN. Bands from the H_trans_-like state are colored in orange, those from the H_inact_-like state obtained under anaerobic conditions in light green and those from the H_inact_-like state under air in dark green. The single and double asterisk indicate HCN and [Fe(CN)_6_]^4−^ respectively. In (A), the additional bands (not colored) correspond to the H_ox_ and H_red_H^+^ states, with small contributions from the H_ox_–CO and H_red_ states. A small proportion of the H_trans_-like state is often present even in freshly-maturated *Dd*HydAB. In (C), the gray bands belong to the H_ox_–CO state. In (D), the small blue-shift observed when the enzyme is exposed to oxygen after addition of NaCN and HAR (H_inact_-like air) might be caused by oxidation of the F-clusters not already oxidized by HAR. As shown in (E), the same “H_inact_-like air” state is routinely present as a minor component when the H_inact_ state (not colored) is prepared by addition of Na_2_S and HAR,^2^ as a result of the oxidation of the H_trans_-like state formed during artificial maturation (shown in A). In (F), the additional bands belong to the H_red_ state, with smaller contributions from the H_red_H^+^ and H_sred_H^+^ states. The H_red_ and H_sred_H^+^ states are present also in (G), after addition of CN^−^ to *Cr*HydA1. Addition of CN^−^ to *Cr*HydA1 seems to induce a lot more H-cluster degradation, as demonstrated by the loss in intensity in the IR spectra (while the protein concentration was similar, 420 μM in (F) and 500 μM in (G)) and the presence of an intense band for [Fe(CN)_6_]^4−^.

Can these CN^−^-dependent states only be formed in [FeFe] hydrogenases with a disrupted proton transfer pathway? IR spectra of artificially maturated WT *Dd*HydAB quite often show a band at 1987 cm^−1^,^45^ which has previously been difficult to assign to any known state of the H-cluster (Fig. 7A). However, we note that the vibrational frequency of this band is similar to the one of the terminal CO ligands in the CN^−^-dependent H_trans_-like state in *Dd*HydAB C178A (1989 cm^−1^) (Fig. 2). Indeed, addition of exogenous CN^−^ to WT *Dd*HydAB caused an increase in intensity of this band, together with the appearance of other bands characteristic of the CN^−^-dependent H_trans_-like state (Fig. 7B).

Oxidation with HAR under anaerobic conditions gives rise to multiple bands in the IR spectrum, some belonging to the H_ox_–CO state and others similar to the H_inact_-like state of the C178A mutant. Subsequent exposure of the enzyme to oxygen, yields a simpler IR spectrum with only an H_inact_-like state present (slightly blue-shifted compared to the analogous state observed under anaerobic conditions, potentially reflecting a difference in the oxidation state of F-clusters, fully oxidized under aerobic conditions) (Fig. 7D). This same CN^−^-dependent H_inact_-like state is present in small amounts when WT *Dd*HydAB is prepared in the H_inact_ state by addition of sulfide,^2^ and probably derives from the small amount of the CN^−^-dependent H_trans_-like state that forms during artificial maturation of this enzyme. Addition of exogenous CN^−^ to WT *Cr*HydA1 also induces formation of a CN^−^-dependent H_trans_-like state (Fig. 7F and G). However, we noted that in this case addition of excess CN^−^ caused substantial degradation of the H-cluster (more than with the *Cr*HydA1 C169A mutant or WT *Dd*HydAB). Therefore, we did not investigate the formation of the H_inact_-like state in WT *Cr*HydA1 in more detail.

## Discussion

In this work, we identified two new H-cluster redox states with electronic structures similar to those of the H_trans_ and H_inact_ states.^2,11,29^ These states have been characterized in detail *via* a combination of spectroscopic, crystallographic and computational techniques. We revealed that the H_trans_-like and H_inact_-like states form upon reaction of [FeFe] hydrogenases with CN^−^. Isotope labelling experiments and X-ray crystallography, supported by computational calculations, suggest that CN^−^ binds at the open coordination site of the H-cluster and, therefore, protects it from O_2_ binding, as it blocks the vacant site. Unlike typical [FeFe] hydrogenase inhibitors such as CO and H_2_S, which bind to the H-cluster reversibly,^2,57^ CN^−^ binding appears to be irreversible, at least under the conditions studied here (pH 8). As noted earlier, purging the *Dd*HydAB C178A variant with CO had no effect (Fig. S1B†) and all samples are initially prepared under an atmosphere of 2% H_2_, suggesting that neither CO nor H_2_ can effectively compete off CN^−^. Although CN^−^ binding to the H-cluster confers air-stability to [FeFe] hydrogenases, the irreversible nature of CN^−^ binding does not make the formation of the H_inact_-like state a suitable strategy to protect [FeFe] hydrogenases during aerobic handling, in contrast to the reversible formation of the H_2_S-dependent H_inact_ state.^2,58^

For the Cys-to-Ala variants and, to a smaller extent, also for the artificially maturated WT *Dd*HydAB, the H_trans_-like state could form even in the absence of exogenous CN^−^. We suggest that the source of CN^−^ in this case derives from the degradation of the [2Fe]_H_ synthetic cofactor during artificial maturation. Degradation of the [2Fe]_H_ cofactor leads to the dissociation of the CO and CN^−^ ligands, which can in turn bind to the ‘intact’ H-clusters. This process of cofactor “cannibalization” is a well-known source of the H_ox_–CO state in WT enzymes,^29^ but this is the first time that CN^−^ binding is also observed. The reason why *Dd*HydAB enzymes (mutant and WT) form more of the H_trans_-like state compared with *Cr*HydA1 is because this enzyme requires longer maturation times with a large excess of cofactor and higher temperature (see Materials and methods in the ESI†), therefore, promoting degradation of the [2Fe]_H_ cofactor and allowing the accumulation of more free CN^−^ in solution. In contrast, artificial maturation of *Cr*HydA1 can be achieved in one hour with only a small excess of the [2Fe]_H_ cofactor. Nevertheless, *Dd*HydAB shows an unusually high affinity towards strong-field ligands, *i.e.* CO and CN^−^, with a tendency to stabilize them in the apical position of the distal Fe ion, as is well known for the CO-inhibited state H_ox_–CO in native *Dd*HydAB. For example, Goldet *et al.* showed that *Dd*HydAB had a 25-fold higher *K*_I_ (inhibition constant for CO during H_2_ oxidation) than *Cr*HydA1, and a 330-fold higher *K*_I_ than HydA1 [FeFe] hydrogenase from *Clostridium acetobutylicum* (*Ca*HydA).^57^ In addition, the C169A variant of *Cr*HydA1,^23,41,44^ the C299A variant of *Cp*HydA1 ^36^ and the C298A variant of *Ca*HydA^59^ have been produced and studied before. In none of these cases was spontaneous formation of CN^−^-bound states observed during artificial maturation, further highlighting that the C178A variant of *Dd*HydAB had especially high affinity for CN^−^. The H_inact_-like crystal structure presented in this work (Fig. 4) at atomic resolution, shows a diatomic ligand at the apical position on Fe_d_, which we have modelled as CN^−^. The reason for this high affinity of *Dd*HydAB towards strong-field ligands is not well understood and needs to be investigated further. Very recently, Duan *et al.* showed binding of CN^−^ to WT *Cp*HydA1 and *Cr*HydA1 using X-ray crystallography and IR spectroscopy.^60^ Their structures show an identical binding mode for the CN^−^ ligand, but the authors propose additional hydrogen bonding interactions from the ADT ligand and nearby cysteine to the nitrogen of the CN^−^ ligand. While their IR spectra of CN^−^-bound WT *Cp*HydA1 appear to be analogous to those from the H_inact_-like states of *Dd*HydAB C178A and *Cr*HydA1 C169A reported here, their IR spectrum of CN^−^-bound *Cr*HydA1 appears to be analogous to our spectrum of *Cr*HydA1 C169A in the H_trans_-like state.

Another example that illustrates the exceptional ligand binding properties of *Dd*HydAB is the formation of the H_2_S-dependent H_inact_ state. Previous work on WT *Dd*HydAB showed that anaerobic oxidation in the presence of sulfide results in binding of SH_2_ to the open coordination site, forming the H_inact_ state, for which the crystal structure revealed a SH^−^ ligand on the apical position of Fe_d_. Sulfide binding to the active site also requires an overoxidized [2Fe]_H_ subcluster (*i.e.* Fe_p_(ii)Fe_d_(ii)).^2,11^ This has been shown to occur in both *Dd*HydAB and *Cr*HydA1,^2^ but much less effectively in *Cp*HydA1, *Ca*HydA and *Me*HydA from *Megasphaera elsdenii*.^34^ Interestingly, during electrochemistry *Dd*HydAB inactivates with a slightly more negative potential (*E*_switch_) than *Cr*HydA1 suggesting that H_2_S binding is faster and H_2_S release is slower for *Dd*HydAB compared with *Cr*HydA1.^2^

In both enzymes, *Dd*HydAB and *Cr*HydA1, the Cys-to-Ala mutation in the proton transfer pathway favors the formation of the CN^−^ bound states. We hypothesize that this may be related to the fact that the H-cluster in the Cys-to-Ala variants seems to be electron-deficient compared to the WT enzymes, as suggested by the blue-shifted bands observed in the IR spectra. This is also in line with previous reports on the altered H_ox_/H_red_ thermodynamics in *Cr*HydA1 C169S.^22^ In contrast, in the HydA1 [FeFe] hydrogenase from *Clostridium acetobutylicum*, the Cys mutation in the PTP to the ionizable residue aspartic acid seems to favor formation of H_ox_ over H_red_.^40^

Why does CN^−^ binding favor H_trans_-/H_inact_-like states? While CN^−^ is generally considered a good π-acceptor, recent experimental and theoretical studies have shown that it is dominated by σ-donating properties with only weak π-accepting properties.^61,62^ Therefore, CN^−^ might stabilize higher oxidation states (*e.g.* the overoxidized [2Fe]_H_ subcluster in the H_trans_-like and H_inact_-like states [Fe_p_(ii)Fe_d_(ii)]_H_) relative for example to CO, which is a better π-acceptor than CN^−^. According to the IR spectra, the Cys-to-Ala variants appear more electron-deficient and, therefore, they have higher affinity for CN^−^ compared to CO. Although we note that the interaction between the cysteine in the PTP and the H-cluster is crucial to modulate the electronic structure at the active site, the detailed understanding of how the Cys-to-Ala mutation affects the electronic distribution at the H-cluster is beyond the scope of this work. However, we suggest that this mutation affects the hydrogen-bonding network surrounding the H-cluster, in particular concerning the ADT amine which has been shown to be involved in electronic delocalization from the two Fe ions in [2Fe]_H_ model compounds.^63^ The reduced steric hindrance in the Cys-to-Ala mutants might also provide easier access to the open coordination site for the additional CN^−^ ligand.

Interestingly, during the synthesis of the [2Fe]_H_ precursor, only two of the CO ligands in Fe_2_[(SCH_2_)_2_NH](CO)_6_ can be substituted with CN^−^.^64^ This shows how the protein scaffold plays a crucial role in stabilizing an additional CN^−^ bound to [2Fe]_H_. One reason could be that within the H-cluster, electron transfer from [2Fe]_H_ to [4Fe-4S]_H_ allows formation of an overoxidized binuclear site, [Fe_p_(ii)Fe_d_(ii)]_H_, which is necessary for CN^−^ binding.

Our report of CN^−^ binding to [FeFe] hydrogenases also sheds light on the nature of previously uncharacterized active-site states. We have demonstrated that the unknown states present as impurities in artificially maturated samples of WT *Dd*HydAB are indeed CN^−^ bound states caused by the long artificial maturation of this enzyme. Previous EPR studies on *Cr*HydA1 C169S have shown the formation of an unidentified H_trans_-like state, exhibiting almost identical *g*-values to the one studied here.^21,22,43^ Thus, it is also plausible that the previously observed H_trans_-like state is caused by binding of CN^−^ to the H-cluster, favored by the Cys-to-Ser mutation. A recent study reported the accumulation over a long time-scale (24 h) of this very similar H_trans_-like state for both the C169S variant and WT *Cr*HydA1 artificially maturated inside *E. coli* cells, leading to the inhibition of H_2_ production by the culture.^43^ We hypothesize that this H_trans_-like state is also CN^−^-dependent as the one described here.

Cyanide binding to metals in biology is well known, with the most classic example being cytochrome c oxidase of the mitochondrial respiratory chain, where CN^−^ binds between heme a_3_ and the Cu_B_ site.^65,66^ Interestingly, in the reduced structure, with Fe(ii) the Fe–C distance is 2.4 Å (ref. 65) and shortens to 2.0 Å in the oxidized structure, with Fe(iii),^66^ suggesting a shorter stronger bond. Cyanide has also been reported to bind ferric heme-proteins with a very high affinity, *e.g.* myoglobin^67–69^ and hemoglobin.^70,71^ In [NiFe] hydrogenase, CN^−^ is thought to bind transiently to the Ni(ii) in the Ni–SI_a_ state, promoting oxidation to Ni(iii) and formation of the Ni–B state.^72^ In CODH, CN^−^ binds again to a Ni(ii) ion with a 1.8 Å Ni–C bond,^73^ and inhibits CO oxidation rather than CO_2_ reduction suggesting that it also binds favorably to a more oxidized active site. Overall, our results are consistent with literature observations that CN^−^ binds preferentially to more oxidized active sites, or alternatively that binding of CN^−^ favors metal oxidation.

## Conclusions

Here, we have reported for the first time, a detailed spectroscopic and computational characterisation of the binding of CN^−^ to the active site of [FeFe] hydrogenases. CN^−^ binding is clearly favored in the Cys-to-Ala mutants, exemplifying the crucial role of the second coordination sphere of the H-cluster in preventing CN^−^ binding, and reflecting the electronic structure adaptations of the H-cluster environment to facilitate stabilization of a terminal Fe(ii)-hydride species during catalysis. Overall, our studies showed how the interaction between the Cys in the PTP and the ADT in [2Fe]_H_ tunes the electronic structure of the active site, controlling ligand binding at the open coordination site.

## Data availability

Data supporting the findings of this study are available in the article and the associated ESI files. Structural data for *Dd*HydAB C178A have been deposited into the Protein Data Bank (PDB) under the following accession codes: 8BJ7 for *Dd*HydAB C178A in the H_inact_-like state and 8BJ8 for *Dd*HydAB C178A in the H_trans_-like state.†

## Author contributions

Conceptualization: M. A. M., J. A. B. and P. R.-M.; methodology: M. A. M., K. B., Y. P., C. L., N. B., I. S., R. B.; investigation: M. A. M., K. B., Y. P., C. L., C. W.; writing – original draft: M. A. M., J. A. B and P. R.-M; writing & editing: all authors; supervision: M. A. M., I. S., R. B., I. Z., S. D., J. A. B and P. R.-M.; funding acquisition: J. A. B, P. R.-M., S. D., I. S., I. Z.

## Conflicts of interest

There are no conflicts to declare.

## Supplementary Material

SC-014-D2SC06098A-s001
