# Supplementary material for: Binding of exogenous cyanide reveals new active-site states in [FeFe] hydrogenases
Source: Chem Sci. 2023 Feb 8;14(11):2826–38. doi: 10.1039/d2sc06098a (PMC10016341; doi:10.1039/d2sc06098a)
Supplement: SC-014-D2SC06098A-s001 [file SC-014-D2SC06098A-s001.pdf]

Electronic Supplementary Material (ESI) for Chemical Science.
This journal is © The Royal Society of Chemistry 2023

Electronic Supplementary Information

Binding of exogenous cyanide reveals new active-site state in [FeFe] hydrogenases

Maria Alessandra Martini[a]*, Konstantin Bikbaev[b], Yunjie Pang[a,c], Christian Lorent[d], Charlotte Wiemann[d,e] Nina Breuer[a], Ingo Zebger[d], Serena DeBeer[a], Ingrid Span[b], Ragnar Bjornsson[a,f], James A. Birrell[a,g]* and Patricia Rodríguez-Maciá[h]*

Affiliations

[a] Department of Inorganic Spectroscopy, Max Planck Institute for Chemical Energy Conversion, Stiftstraße 34-36, 45470 Mülheim an der Ruhr, Germany.

[b] Department of Chemistry and Pharmacy, Bioinorganic Chemistry, Friedrich Alexander University Erlangen-Nürnberg, Erlangen, Germany.

[c] College of Chemistry, Beijing Normal University, 100875, Beijing, China

[d] Institut für Chemie, Technische Universität Berlin, Straße des 17. Juni 135, 10623 Berlin, Germany.

[e] Ruanda-Zentrum und Büro für Afrika-Kooperationen, Universität Koblenz-Landau, Universitätsstraße 1, 56070 Koblenz

[f] Univ. Grenoble Alpes, CNRS, CEA, IRIG, Laboratoire de Chimie et Biologie des Métaux, 17 Rue des Martyrs, F-38054 Grenoble, Cedex, France.

[g] School of Life Sciences, University of Essex, Colchester, CO4 3SQ, UK

[h] Department of Chemistry, Inorganic Chemistry Laboratory, University of Oxford, South Parks Road, Oxford, OX1 3QR, UK

Contents

Materials and Methods 3
Supplementary Tables 6
Supplementary Figures 8
Supplementary References 20

Materials and Methods

Protein sample preparation

The *Dd*HydAB C178A variant was generated by site directed mutagenesis using PCR with non-overlapping primers (forward primer: <u>*GC*</u>CTGTCCGGGTTGGCAA, reverse primer: AGACGTGAACTGCGGCA) in which the forward primer contained the mutagenic codon at the 5' end. Following PCR, template DNA was digested with DpnI restriction endonuclease, the blunt ended PCR product was gel-purified, circularized using T4 polynucleotide kinase and T4 DNA ligase, and used for transformation of NEB 10β competent *E. coli* cells, which were plated on LB-agar containing 35 µg mL^{-1} chloramphenicol. Single colonies were used to inoculate LB containing 35 µg mL^{-1} chloramphenicol and grown overnight at 37 ^{o}C. Cells were then harvested, DNA was extracted, purified and sent for sequencing.

WT and C169A *Cr*HydA1[1], WT and C178A *Dd*HydAB were recombinantly expressed in *E. coli* BL21(DE3) Δ*iscR* as Strep-tagII fusion proteins in their apo-form (i.e. lacking the $[2Fe]_H$ subcluster) and purified by affinity chromatography, as previously described[2, 3] (but without co-expression of the maturases). The $[2Fe]_H$ precursor $(Et_4N)_2[Fe_2(ADT)(CO)_4(CN)_2]$ was synthesized according to literature procedures.[4] Protein samples were artificially maturated with the $[2Fe]_H$ precursor as described.[3, 5] Briefly, for the artificial maturation apo-hydrogenases diluted in 100 mM Tris pH 8, 150 mM NaCl were mixed with $(Et_4N)_2[Fe_2(ADT)(CO)_4(CN)_2]$ (2 eq. for *Cr*HydA1, 5 eq. for *Dd*HydAB) and incubated at room temperature (RT) for 1 h (*Cr*HydA1), or at 35 °C for 50 h (*Dd*HydAB). Samples were then buffer exchanged to 25 mM Tris pH 8, 25 mM KCl to remove excess of $[2Fe]_H$ precursor using a Sephadex G-25 desalting column. WT *Dd*HydAB required an additional incubation (>48 h at RT) in an open vessel to allow re-activation of the CO-inhibited state.[3] Protein maturation and handling were performed under dim light due to light-sensitivity of these enzymes (*Dd*HydAB in particular).[6] Concentrated protein samples were stored in gas-tight vials at -80 °C until use. As maturated *Cr*HydA1 C169A slowly forms the H_{trans}-like state over time, samples of this variant were used within one week of preparation. Sodium dithionite (NaDT) was excluded from all protein preparations to avoid contamination by its oxidation products.[7]

Preparation of samples for spectroscopic measurements

Protein samples were prepared in 25 mM Tris pH 8, 25 mM KCl under anaerobic conditions (2% H_2 in 98% N_2) or under air where specified. NaCN (1-5 mM), hexaammineruthenium (III) chloride (HAR) (100 µM -10 mM) and NaDT (10 mM) were added where specified in figures and captions. For the preparation of *Cr*HydA1 C169A in the H_{trans}-like state, 200 µM protein was mixed with 100 µM hexammineruthenium (III) (HAR) and 5 mM NaCN (or $K^{13}CN$) and then buffer exchanged to 25 mM Tris pH 8, 25 mM KCl on a Sephadex G-25 desalting column to remove excess CN^- and H-cluster degradation products. WT *Dd*HydAB was prepared in the H_{inact} state as previously described.[8]

IR Spectroscopy

IR spectra were recorded on a Bruker Vertex 80v FT-IR spectrometer equipped with a mercury cadmium telluride (MCT) detector cooled by liquid nitrogen. Samples were placed between CaF_2 windows separated by a Teflon spacer (50 µm) and sealed in home-built brass holders with rubber rings. Spectra were recorded in the double-sided, forward-backward mode, with a resolution of 2 cm^{-1}, an aperture setting of 3 mm and scan velocity of 20 kHz. FTIR data were processed using home-written routines in the MATLAB environment.

Cryogenic IR spectra (Figure S10) were recorded on a Bruker Tensor 27 FT-IR spectrometer linked to a Hyperion 3000 IR microscope equipped with a 20× IR transmission objective and a mercury cadmium telluride (MCT) detector as previously described.[9] The temperature was set to 80 K by a liquid-N_2-cooled cryo-stage (Linkam Scientific instruments). Illumination was performed using the focused beam of a

collimated 530 nm LED with a power density of ca. 500 mW cm^{-2}. Data were processed using the OPUS software version 7.5 from Bruker.

EPR spectroscopy

For X-band EPR spectroscopy, samples (~200 µL) were transferred to X-band quartz EPR tubes and frozen in liquid N_2. In parallel, one aliquot of each sample was measured by IR spectroscopy at room temperature. Spectra were recorded on a Bruker ELEXSYS E500 CW X-band EPR spectrometer. The temperature was controlled with an Oxford ESR900 helium flow cryostat. Measurement parameters: modulation frequency 100 kHz; modulation amplitude 7.46 Gauss; time constant 81.92 ms; conversion time 81.92 ms. Temperature and microwave power were varied and are specified in figure legends. Spectra were processed using home-written routines in the MATLAB environment. Spectral simulations were performed with EasySpin package[10] in MATLAB.

X-ray crystallography

The *Dd*HydAB C178A protein was buffer exchanged into 10 mM Tris-HCl at pH 7.6 using a Sephadex G-25 desalting column prior to crystallization. Crystals were obtained using the sitting drop vapor diffusion method in 1 M lithium chloride, 0.1 M sodium acetate, and 30 % polyethylene glycol 4000 with a protein:precipitant ratio of 1:1. The *Dd*HydAB C178A H_{inact}-like state crystallized at 8 °C under aerobic conditions in the dark. We used a protein concentration of 25 mg/mL and a drop size of 2.0 µL. Crystallization of *Dd*HydAB C178A H_{trans}-like state was performed at 22 °C under anaerobic atmosphere (2% H_2 and 98% N_2 in a vinyl anaerobic chamber) in the dark. The protein concentration of the sample was 35 mg mL^{-1} and the drop size was 2.2 µL. Crystals were harvested using MicroMounts or MicroLoops after three days, transferred into a cryo-protectant solution consisting of 50% (w/v) aqueous polyethylene glycol 4000 and stored in liquid N_2. Datasets were collected at an energy of 15 keV and 100 K at PETRA III, beamline P11, Deutsches Elektronensynchrotron (DESY, Hamburg, Germany). Data were processed using XDS[11] and data reduction was performed with AIMLESS[12] within the CCP4i2[13] suite. Molecular replacement was performed using Phaser[14] within the CCP4i2 suite with the coordinates of PDB ID 6SG2 as starting model. Structure refinement, model building, and validation were performed using REFMAC,[15] Coot and CCP4i2. The electron density maps were calculated using FFT[16] in CCP4 and the omit map was calculated with REFMAC using a model lacking the [2Fe] subcluster and additional CN^- ligand. PyMOL was used to prepare the figures and to calculate the root-mean-square deviation (RMSD) of the Cα atoms of residues 2-397 using the align command with the number of cycles set to 0, thus, not including outlier rejection. The final models of DdH C178A in both states contained 98 % in the favored region, 2 % in the allowed region, and 0 % in outlier regions of the Ramachandran plot as defined by MolProbity.[17] The atomic coordinates have been deposited with the Protein Data Bank, Research Collaboratory for Structural Bioinformatics at Rutgers University (PDB ID: 8BJ7 for *Dd*HydAB C178A in the H_{inact}-like state and 8BJ8 for *Dd*HydAB C178A in the H_{trans}-like state).

Single crystal infrared microspectroscopy

Crystals from a batch of crystals of *Dd*HydAB C178A obtained under aerobic conditions were collected, transferred to homemade MgF_2 plates and immediately frozen in liquid nitrogen. The same crystals were later analyzed by resonance Raman spectroscopy (see below). IR spectra were recorded on a Bruker Tensor 27 FT-IR spectrometer linked to a Hyperion 3000 IR microscope equipped with a 20× IR transmission objective and a mercury cadmium telluride (MCT) detector as previously described. The

temperature was set to 233 K utilizing a liquid-N_2-cooled cryo-stage (Linkam Scientific instruments). The raw data were processed using the OPUS software version 7.5 from Bruker.

QM/MM calculations

The quantum mechanics/molecular mechanics (QM/MM) model of the *Dd*HydAB C178A variant was based on a previous wild-type model[18] with the mutation Cys178 to Ala added. The minimum QM region defined included the $[2Fe]_H$ subcluster, the Ala178 amino acid close to the ADT ligand, and Cys382, the cysteine bridging $[2Fe]_H$ and $[4Fe\text{-}4S]_H$ (see Figure S3). The $[4Fe\text{-}4S]_H$ and the additional coordinating cysteine residues were added to the minimum QM region to obtain a larger model (named minimum + $[4Fe\text{-}4S]_H$). A larger (medium) model included 12 more residues to account for hydrogen bonding around the $[2Fe]_H$: Ala109, Pro108, Ala107, Ser202, Pro203, Ile204, Met232, Lys237, Gly292, Ala293, Thr294, and Sol-2427 (water molecule), shown in Figure S3.

Calculations were performed with the QM/MM code, ASH, developed by R. Björnsson.[19] ASH has an interface to the OpenMM molecular mechanics library and the ORCA quantum chemistry code. Standard electrostatic embedding with linkatoms and charge-shifting was used in the QM/MM calculations. The MM part were described using a modified CHARMM36 force-field[20] (with a simple non-bonded model for the metal clusters) with periodic boundary conditions enabled. The ORCA code[21] was utilized for the QM calculations. The r^2SCAN[22] density functional was used for the QM-part which has been shown to work well for both open-shell and closed-shell Fe-S dimers according to a recent benchmarking study.[23] The Split-RI-J approximation[24] and with a decontracted auxiliary basis set[25, 26] (named SARC/J in ORCA) was used to speed up the Coulomb integral evaluations. The ZORA scalar relativistic Hamiltonian[27, 28] was used with relativistically recontracted basis sets: the ZORA-def2-TZVP[25, 29] basis set was used on $[2Fe]_H$, the extra ligand on Fe_d, and the $[4Fe\text{-}4S]_H$ cubane (when included in the QM-region), and other atoms were calculated with ZORA-def2-SVP. The D4 dispersion correction[30, 31] was used in all calculations. A partial Hessian approach,[32] was used to calculate numerical vibrational frequencies, with two Fe ions, all CO, and all CN^- groups included in the definition of the partial Hessian. IR intensities and regular Raman activities (polarizability derivatives) were calculated using ORCA.

Resonance Raman spectroscopy

Small aliquots of *Cr*HydA1 C169A in the H_{trans}-like state (2.5 µL, 4 mM) were transferred to quartz plates and frozen in liquid nitrogen under an anaerobic atmosphere. For isotopic labelling experiments the samples were prepared with natural abundance NaCN and ^{13}C-labeled KCN (see IR spectra in Figure 6) then exposed to air for 5 min at 10 °C to form the H_{inact}-like state and frozen. Resonance Raman spectra were recorded on a LabRam HR-800 Jobin Yvon confocal Raman spectrometer connected to a liquid-N_2-cooled charge-coupled device as previously described.[9, 33] The 514 nm emission line of an Ar^+ -ion laser with 2 mW power was used for excitation. Immediately before measurements, the sample was thawed at 10 °C and exposed to atmospheric oxygen for 5 minutes to form the H_{inact}-like state, then frozen again, setting the temperature to 80 K by using by a liquid-N_2-cooled cryo-stage (Linkam Scientific instruments). Data were processed using OPUS software version 7.5 from Bruker.

Supplementary Tables

Table S1 – IR bands of relevant [FeFe] hydrogenase states observed in this and previous studies

Enzyme	State	CN	t-CO	µ-CO	Ref
*Dd*HydAB C178A	H_{trans}-like (CN^-)	2116; 2100; 2087	1989	1853	This work
	H_{trans}-like (CN^-) F_{red}	2114; 2098; 2085	1986	1838	This work
	H_{inact}-like (CN^-)	2126; 2108; 2098	2017; 1994	1870	This work
WT *Dd*HydAB	H_{ox}	2089; 2079	1965; 1940	1802	[34]
	$H_{red}H^+$	2078; 2041	1915; 1894	1810	[35]
	H_{ox}-CO	2096; 2089	2017*; 1972; 1963	1812	[34]
	H_{trans}	2100; 2075	1988; 1976	1835	[8]
	H_{inact}	2106; 2086	2007; 1983	1847	[8]
*Cr*HydA1 C169A	H_{red}-like†	2089; 2068	1971; 1939	1804	This work
		2088; 2072	1970; 1938	1804	[36]
	H_{hyd}	2089; 2076	1980; 1962	1864	This work
		2082; 2068	1978; 1962	1862	[36]
	H_{trans}-like (CN^-)	2114; 2097; 2082;	1988	1856	This work
	H_{inact}-like (CN-) (anaerobic)	2122; 2104; 2090	2012; 1996	1868	This work

†This state was previously assigned to be H_{ox}. We suggest that this state to actually have an electronic structural similar to H_{red} (i.e. with ($[4Fe\text{-}4S]_H^{+}$-$[Fe_p(II)Fe_d(I)]_H$).

*In the H_{ox}-CO state the two CO ligands bound to Fe_d are strongly vibrationally coupled, giving rise to a higher energy symmetric stretching band and a lower energy antisymmetric stretching band.

Table S2 – Data collection and refinement statistics

	HydAB C178A H_{inact}-like	HydAB C178A H_{trans}-like
**Data collection**		
Space group	$P2_12_12_1$	$P2_12_12_1$
Cell dimensions		
a, *b*, *c* (Å)	49.328, 87.281, 88.977	49.387, 87.411, 89.125
α, β, γ (°)	90.00, 90.00, 90.00	90.00, 90.00, 90.00
Resolution (Å)	44.49 - 1.04 (1.06 – 1.04)	44.56 - 1.01 (1.03 – 1.01)
R_{merge}	0.199 (2.447)	0.087 (2.148)
$I / \sigma I$	10.9 (1.1)	13.9 (0.9)
Completeness (%)	99.7 (99.9)	99.3 (93.4)
Redundancy	13.0 (12.0)	12.6 (8.7)
**Refinement**		
Resolution (Å)	44.49 - 1.04 (1.06 – 1.04)	44.56 - 1.01 (1.03 – 1.01)
No. reflections	2379468 (108100)	2520730 (80133)
R_{work} / R_{free}	0.158 / 0.167	0.151 / 0.164
No. atoms		
Protein	7189	7113
Ligand/ion	50	50
Water	440	465
B-factors		
Protein	12.9	12.2
Ligand/ion	8.9	8.0
Water	22.6	21.0
R.m.s. deviations		
Bond lengths (Å)	0.0142	0.0150
Bond angles (°)	2.144	2.138

Supplementary Figures

A

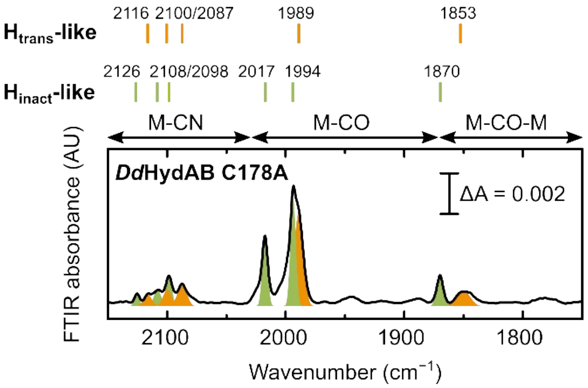

B

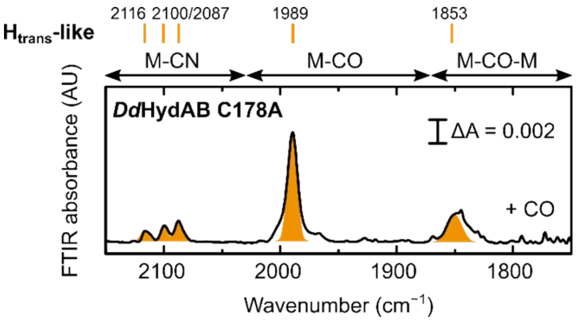

Figure S1. A) IR spectra of *Dd*HydAB C178A after storage at -80 °C. Aliquots of the same sample were used to collect data shown in Figure 2. B) After maturation, *Dd*HydAB C178A was flushed with 100 % CO for 5 min and immediately transferred (under 2 % H_2) to the IR cell. Even after CO treatment, the enzyme remained in the H_{trans}-like state.

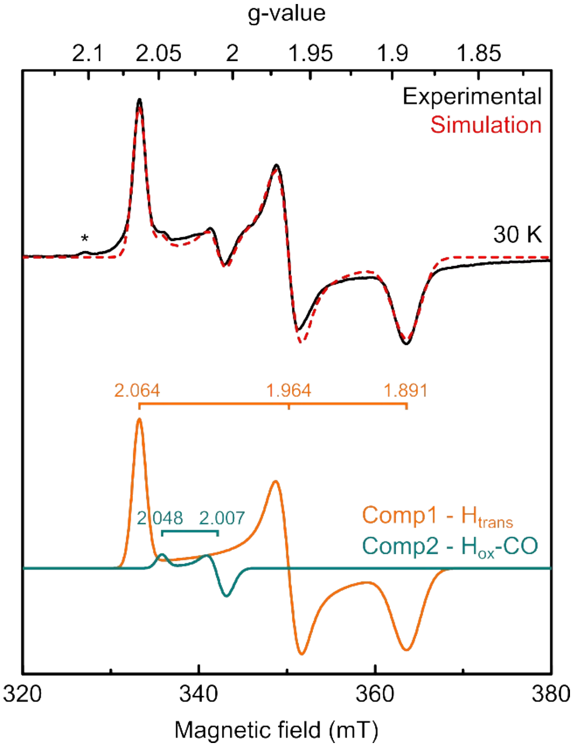

Figure S2. CW X-band EPR spectrum of WT *Dd*HydAB in the H_{trans} state. The H_{trans} state was prepared by treating *Dd*HydAB with 1 mM HAR and 10 mM Na_2S as described previously.[8] The experimental spectra (black) are overlaid with spectral simulations (dashed red lines) and the component spectra are shown underneath. The orange trace (Component 1) corresponds to the H_{trans} state, while the dark cyan trace (Component 2) corresponds to the H_{ox}–CO state. The asterisk indicates a small contribution from the H_{ox} state. The *g* values for the H_{trans} state (*g* = 2.064, 1.964, 1.891) are in excellent agreement with those reported previously by Albracht *et al* (*g* = 2.06, 1.96, 1.89).[37] EPR experimental conditions: microwave frequency = 9.63 GHz; microwave power = 100 mW; temperature = 30 K.

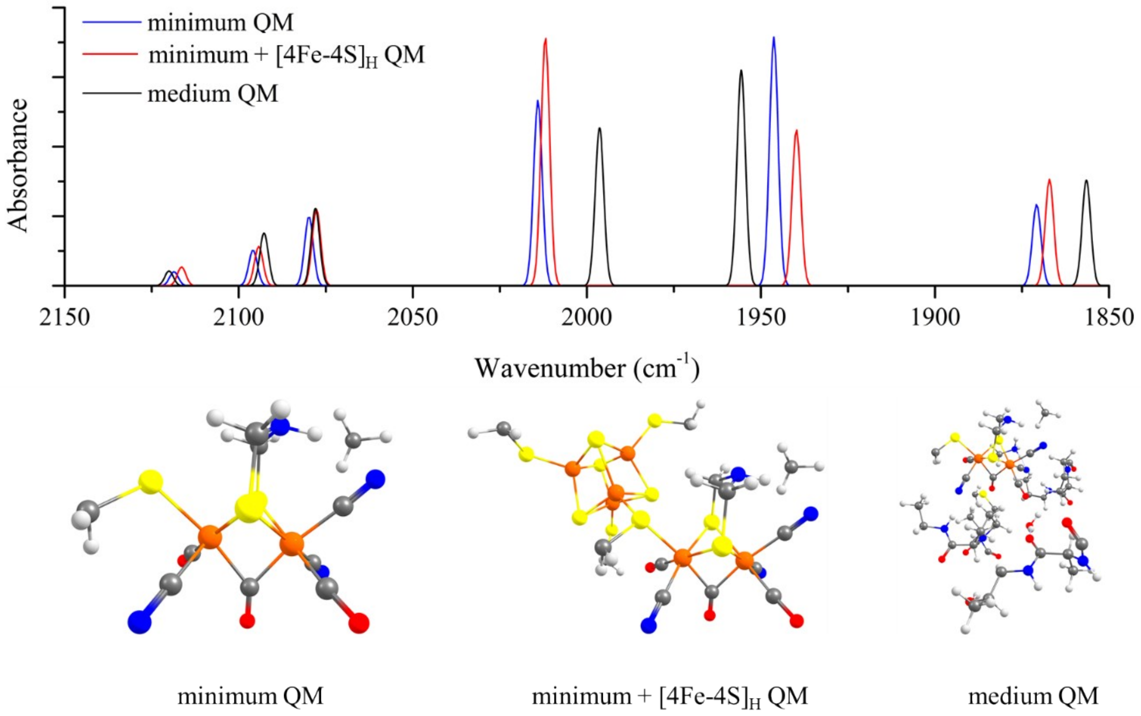

Figure S3. The definition of different quantum mechanical (QM) regions and the effect on calculated IR spectra in the H_{inact} state. The scaling factor for vibrational frequencies is 0.964. Inclusion of the $[4Fe\text{-}4S]_H$ cubane in the QM region (minimum + $[4Fe\text{-}4S]_H$ region) results in very small shifts of the CN^- modes and only slightly larger shifts for the CO modes. The larger QM region ("medium") includes protein residues around the $[2Fe]_H$ to better account for hydrogen bonding: Ala109, Pro108, Ala107, Ser202, Pro203, Ile204, Met232, Lys237, Gly292, Ala293, Thr294, and Sol-2427 (water molecule); calculations with this region results in larger shifts, particularly for the CO modes with the CN modes less affected.

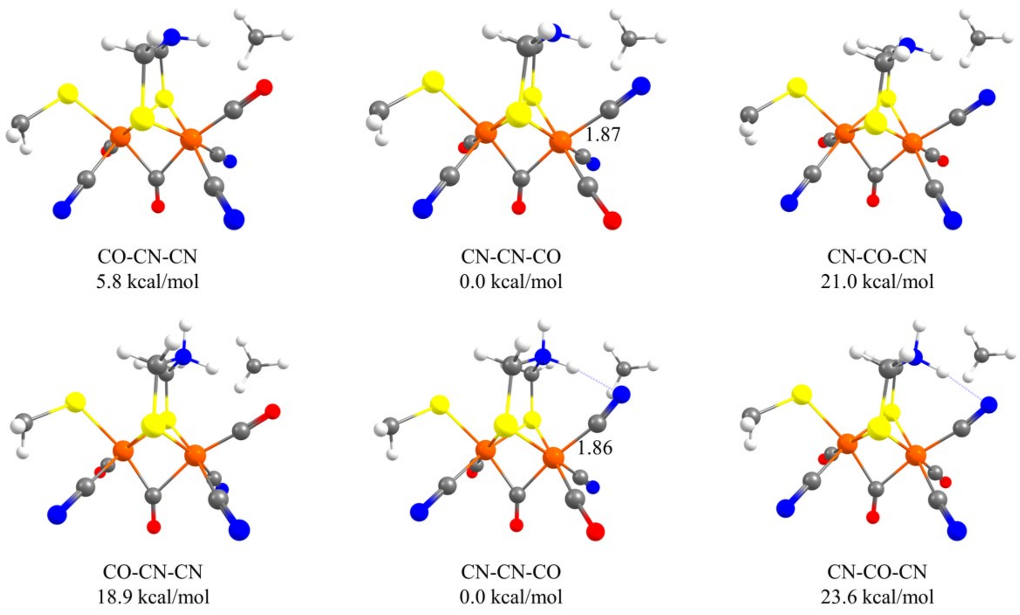

Figure S4. The relative QM/MM energies for different rotameric structures of CN^--bound to Fe_d in the H-cluster models in both ADT (top) and ADTH (bottom) protonation states in the $[Fe(II)Fe(II)]_H$ redox state. Labels refers to the clockwise sequence of the three groups from the exogenous position. The calculations were preformed using the medium QM region (see Figure S3). The model with apical-CN^- on Fe_d with the other CO/CN^- in their regular positions (CN-CN-CO) is the most favorable for both ADT and ADTH models. The trends in energies for can be rationalized by more favorable hydrogen-bonding involving apical CN^- and ADT (especially when protonated). Also, a Lys237 cation (not shown) donates a hydrogen bond with the ligand pointing away from the viewer, which is more favorable with a CN^- ligand in this position (CO-CN-CN and CN-CN-CO models).

A B

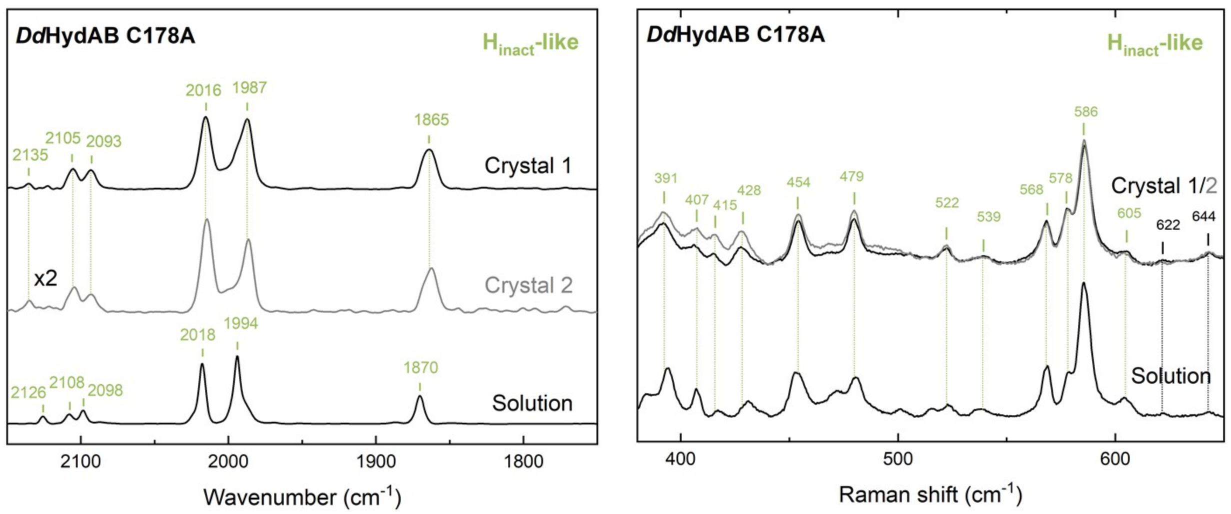

Figure S5. IR and RR spectra of *Dd*HydAB C178A crystals in the H_{inact}-like state. A) IR spectra of *Dd*HydAB C178A crystals prepared under aerobic conditions recorded at 233 K in comparison to the corresponding spectrum in solution measured at 293 K. These data clearly confirm the presence of the H_{inact}-like state in the protein crystals. Small phase-dependent (crystal vs. solution) shifts in the band positions are likely related to temperature-dependent changes or crystal packing effects, which may cause e.g. small rearrangements in the hydrogen-bonding networks. B) Corresponding RR spectra of the same *Dd*HydAB C178A crystals recorded at 80 K via excitation by the 514 nm line of an Ar^+ laser in comparison to the respective spectrum in solution. The assignment of the H_{inact}-like state to the crystal is additionally verified by an almost identical spectral signature in the region of the Fe-CO/CN metal ligand modes of the protein crystals and solution. The spectra were normalized to modes at 622 cm^{-1} and 644 cm^{-1} that correspond to the amino acid side-chain vibrations of phenylalanine and tyrosine, respectively.[38]

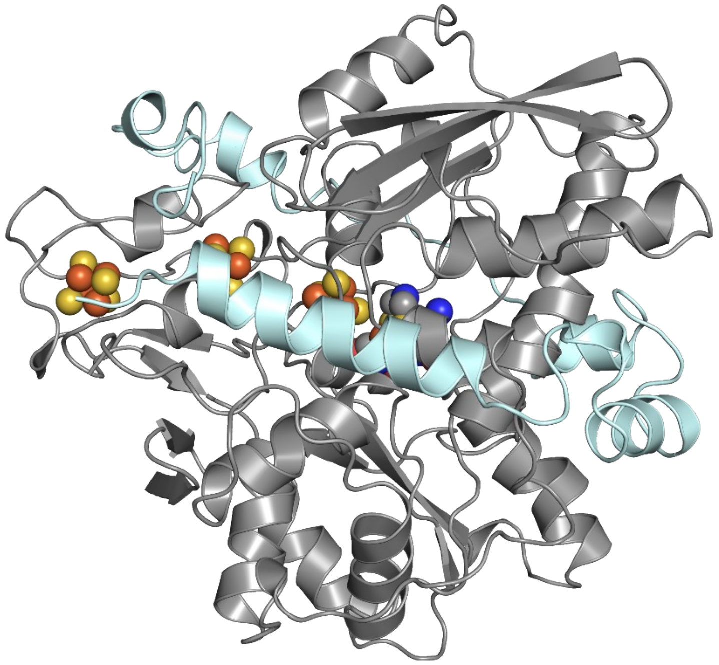

Figure S6. Overall view of *Dd*HydAB C178A H_{inact}-like with schematic representation of the [4Fe-4S]-clusters and H-cluster.

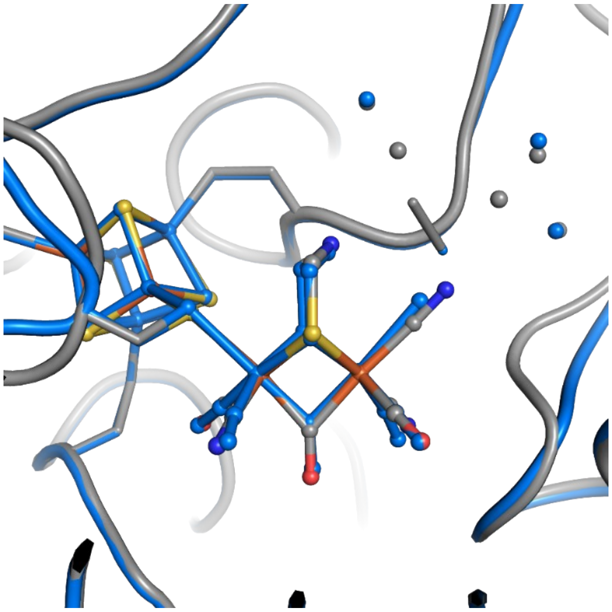

Figure S7. Superposition of the *Dd*HydAB C178A H_{inact}-like structure (gray) with the *Dd*HydAB WT H_{inact} structure (blue) showing a close-up view of the active site.

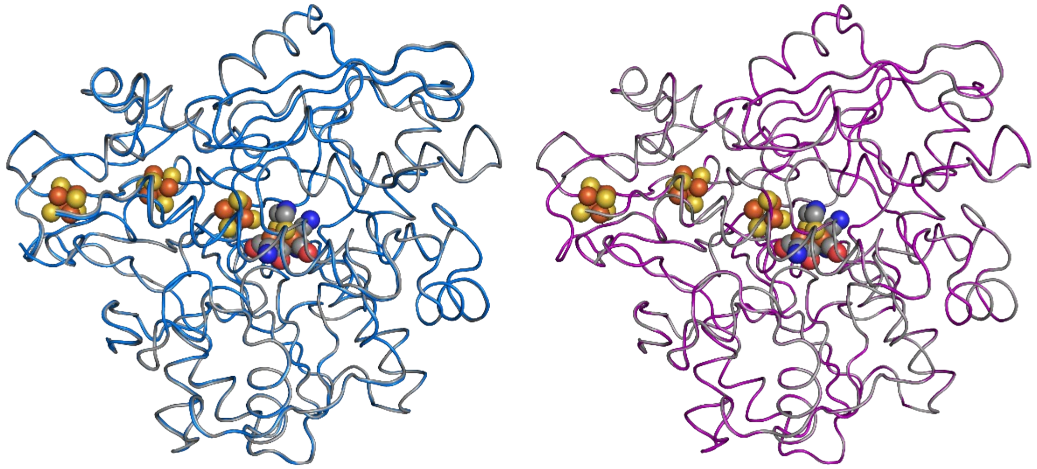

Figure S8. A) Superposition of the *Dd*HydAB C178A H_{inact}-like structure (gray) with the *Dd*HydAB WT H_{inact} structure (blue) and with the *Dd*HydAB C178A H_{trans}-like structure (magenta).

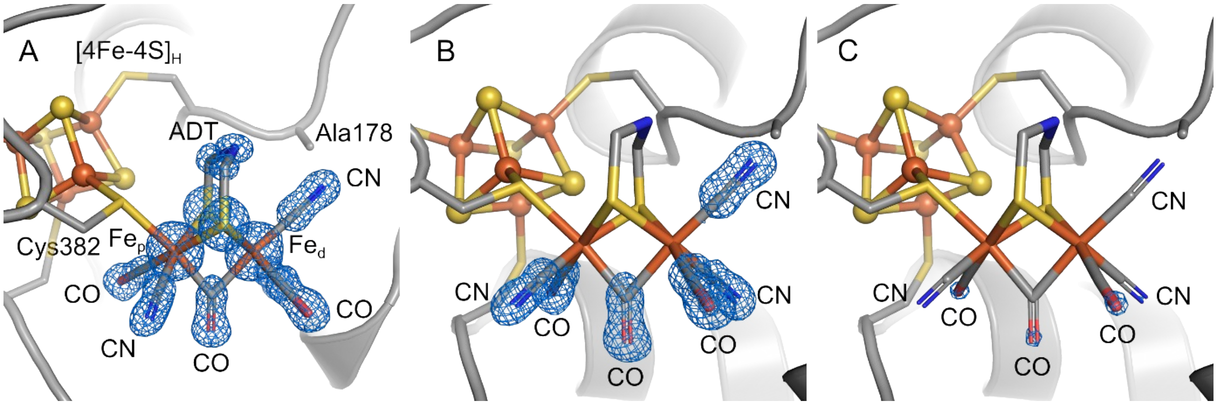

Figure S9. Crystal structure of the *Dd*HydAB C178A variant in the H_{inact}-like state with an omit map calculated for the $[2Fe]_H$ subsite. (A) An omit map (blue mesh, contoured at 1.0 σ) is shown for the $[2Fe]_H$ subsite including the CN^- ligand in the open coordination site. (B) An omit map (blue mesh, contoured at 1.0 σ) is displayed for all CO and CN^- ligands. (C) The same omit map (blue mesh, contoured at 2.8 σ) as displayed in (B) with a different contour level supports the assignment of the nitrogen and oxygen atoms. The protein backbone is represented as cartoon, amino acid side chains and the $[2Fe]_H$ subsite are shown as sticks, $[4Fe-4S]_H$ clusters are shown as ball-and-stick model.

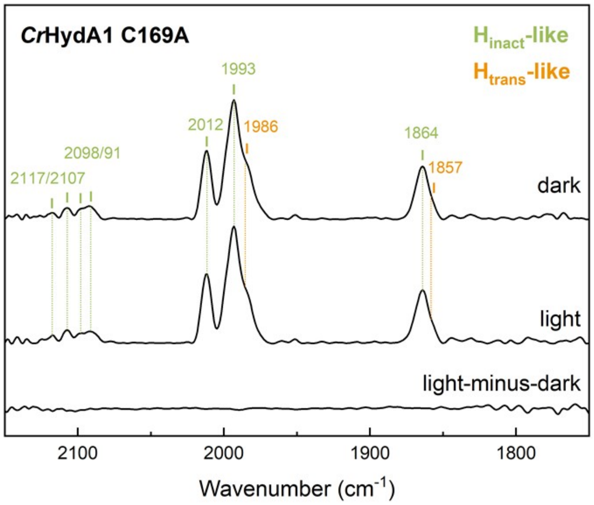

Figure S10. Cryogenic IR spectra of *Cr*HydA1 C169A in the H_{inact}-like and H_{trans}-like state illuminated with blue light. IR spectra were recorded at 80 K in the dark (top), after 20 min illumination by the focused beam of a collimated power LED at 530 nm (middle) and the corresponding light-minus-dark difference spectrum. This illumination experiment revealed no obvious photosensitivity of the H_{trans}-like and H_{inact}-like states, rather excluding the binding of an additional terminal CO.

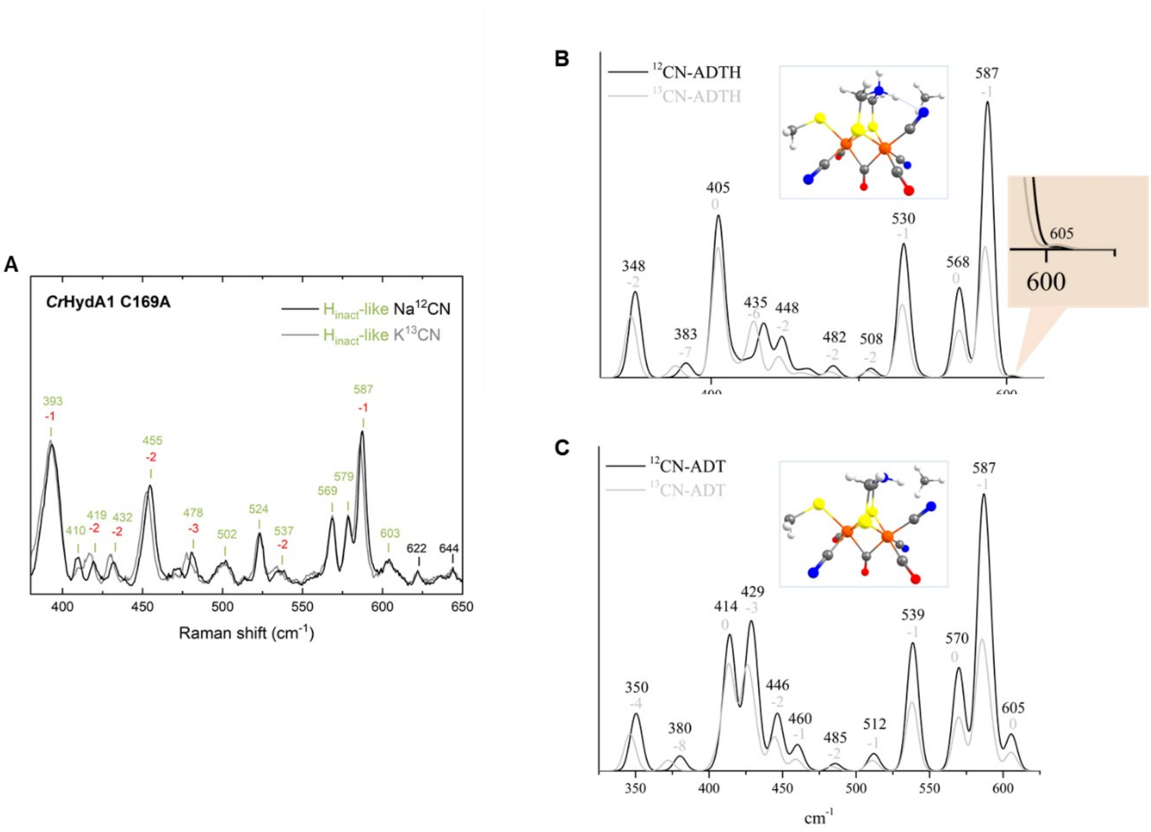

Figure S11. Comparison of experimental and calculated Raman spectra of *Cr*HydA1 C169A, where the CN^- bound apical at Fe_d was isotopically labelled. A) Experimental RR spectra from the natural abundance CN^- and $^{13}CN^-$ in the H_{inact}-like state are displayed in black and gray respectively, with the isotopic shifts from ^{12}C to ^{13}C displayed as red numbers. RR spectra were measured at 80 K using the 514 nm line of an Ar^+ laser for excitation. The small shifts to lower frequency are in line with a slightly weaker metal ligand vibration originating from the heavier ^{13}C atoms bound terminal at the $[2Fe]_H$ subcluster. The spectra were normalized to modes at 622 cm^{-1} and 644 cm^{-1} that correspond to the amino acid side-chain vibrations of phenylalanine and tyrosine, respectively.[38] Calculated Raman spectra, based on the *Dd*HydAB C178A model, for (B) ADTH and (C) ADT models, using the medium QM region. Calculated frequencies were scaled to match the most easily identifiable peak on the experimental spectrum: the intense 587 cm^{-1} peak. This was done by dividing the experimental and corresponding calculated frequencies of the exp. 587 cm^{-1} peak. This resulted in scaling factors of 0.945 (ADTH) and 0.938 (ADT) which were then applied to all frequencies. The mass of the carbon in the apical CN^- ligand was either 12 or 13 u. Regular Raman activities were calculated from polarizability derivatives. The experimental isotope shift on the spectra is overall well reproduced (some overestimation seen). The experimental peak at 603 cm^{-1} is reproduced computationally in the ADT model at 605 cm^{-1} (c) but not with the ADTH model (b). This peak is primarily associated with the bending of the bridging CO, which is likely perturbed to different degrees via a trans-effect relating to different degrees of CN^- binding in ADTH model vs. ADT model. Each vibrational transition was broadened with a Gaussian with a width of 10 cm^{-1} (FWHM). The broad experimental peak at 455 cm^{-1} was calculated as either an intense peak at 405 cm^{-1} (ADTH) or a split peak at 414-429 cm^{-1}.

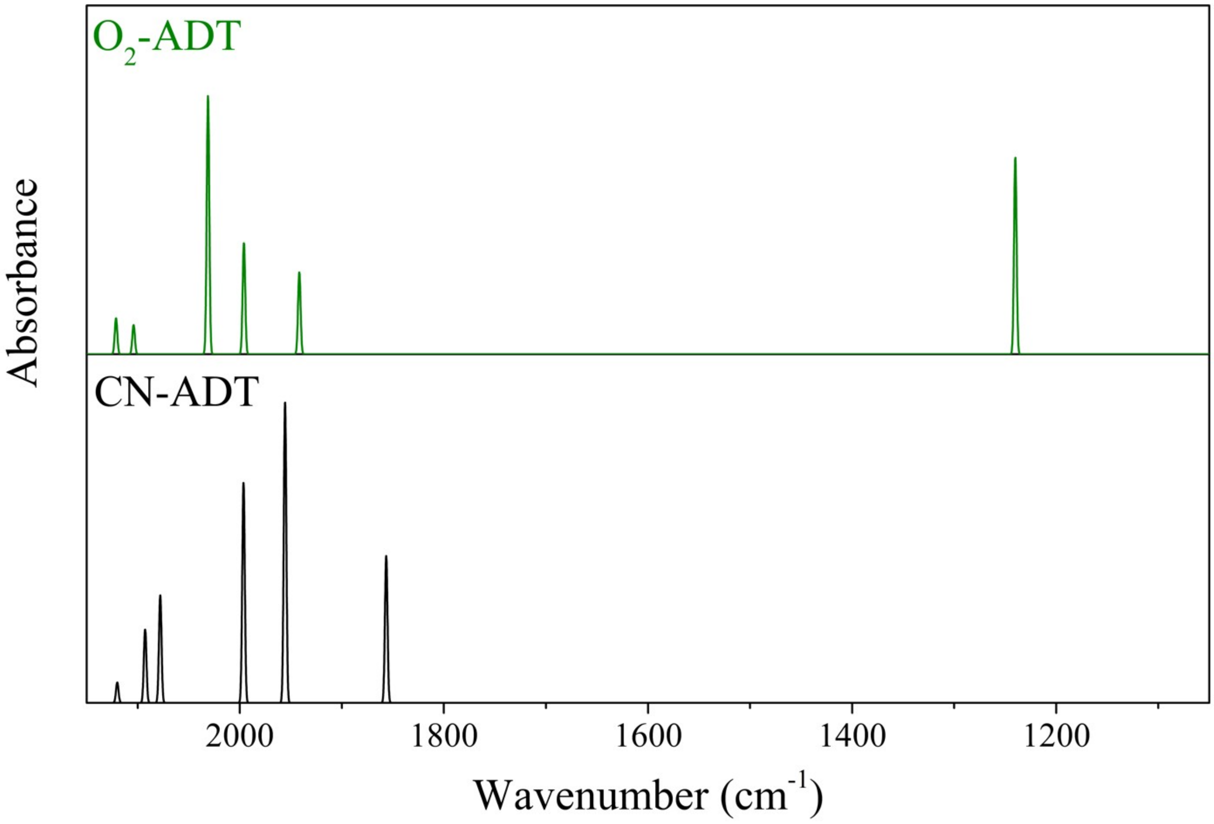

Figure S12. A comparison of calculated IR spectra when the exogenous ligand is modelled as CN^- or O_2 using the QM/MM model with a singly protonated ADT ligand. Frequencies are scaled by 0.964. Optimization of an O_2 model results in spontaneous superoxide formation according to geometry and spin density distribution.

Supplementary References

1. S. Rumpel, C. Sommer, E. Reijerse, C. Fares and W. Lubitz, *J. Am. Chem. Soc.*, 2018, **140**, 3863-3866.
2. J. M. Kuchenreuther, C. S. Grady-Smith, A. S. Bingham, S. J. George, S. P. Cramer and J. R. Swartz, *PLoS One*, 2010, **5**, e15491.
3. J. A. Birrell, K. Wrede, K. Pawlak, P. Rodriguez-Maciá, O. Rüdiger, E. J. Reijerse and W. Lubitz, *Isr. J. Chem.*, 2016, **56**, 852-863.
4. H. Li and T. B. Rauchfuss, *J. Am. Chem. Soc.*, 2002, **124**, 726-727.
5. J. Esselborn, C. Lambertz, A. Adamska-Venkatesh, T. Simmons, G. Berggren, J. Noth, J. Siebel, A. Hemschemeier, V. Artero, E. Reijerse, M. Fontecave, W. Lubitz and T. Happe, *Nat. Chem. Biol.*, 2013, **9**, 607-609.
6. W. Roseboom, A. L. De Lacey, V. M. Fernandez, E. C. Hatchikian and S. P. J. Albracht, *J. Biol. Inorg. Chem.*, 2006, **11**, 102-118.
7. M. A. Martini, O. Rüdiger, N. Breuer, B. Nöring, S. DeBeer, P. Rodríguez-Maciá and J. A. Birrell, *J. Am. Chem. Soc.*, 2021, **143**, 18159-18171.
8. P. Rodriguez-Macia, E. J. Reijerse, M. van Gastel, S. DeBeer, W. Lubitz, O. Rudiger and J. A. Birrell, *J. Am. Chem. Soc.*, 2018, **140**, 9346-9350.
9. C. Lorent, V. Pelmenschikov, S. Frielingsdorf, J. Schoknecht, G. Caserta, Y. Yoda, H. Wang, K. Tamasaku, O. Lenz, S. P. Cramer, M. Horch, L. Lauterbach and I. Zebger, *Angew. Chem. Int. Ed.*, 2021, **60**, 15854-15862.
10. S. Stoll and A. Schweiger, *Journal of Magnetic Resonance*, 2006, **178**, 42-55.
11. W. Kabsch, *Acta Crystallograph. D*, 2010, **66**, 125-132.
12. P. R. Evans and G. N. Murshudov, *Acta Crystallogr. D*, 2013, **69**, 1204-1214.
13. M. D. Winn, C. C. Ballard, K. D. Cowtan, E. J. Dodson, P. Emsley, P. R. Evans, R. M. Keegan, E. B. Krissinel, A. G. W. Leslie, A. McCoy, S. J. McNicholas, G. N. Murshudov, N. S. Pannu, E. A. Potterton, H. R. Powell, R. J. Read, A. Vagin and K. S. Wilson, *Acta Crystallogr. D*, 2011, **67**, 235-242.
14. A. J. McCoy, R. W. Grosse-Kunstleve, P. D. Adams, M. D. Winn, L. C. Storoni and R. J. Read, *J. Appl. Crystallogr.*, 2007, **40**, 658-674.
15. G. N. Murshudov, A. A. Vagin and E. J. Dodson, *Acta Crystallogr. D*, 1997, **53**, 240-255.
16. L. Ten, *Acta Crystallogr. A*, 1973, **29**, 183-191.
17. V. B. Chen, W. B. Arendall, III, J. J. Headd, D. A. Keedy, R. M. Immormino, G. J. Kapral, L. W. Murray, J. S. Richardson and D. C. Richardson, *Acta Crystallogr. D*, 2010, **66**, 12-21.
18. P. Rodriguez-Macia, L. M. Galle, R. Bjornsson, C. Lorent, I. Zebger, Y. Yoda, S. P. Cramer, S. DeBeer, I. Span and J. A. Birrell, *Angew. Chem. Int. Ed.*, 2020, **59**, 16786-16794.
19. R. Björnsson, ASH - a multiscale modelling program, https://ash.readthedocs.io).
20. R. B. Best, X. Zhu, J. Shim, P. E. M. Lopes, J. Mittal, M. Feig and A. D. MacKerell, Jr., *J. Chem. Theory Comput.*, 2012, **8**, 3257-3273.
21. F. Neese, F. Wennmohs, U. Becker and C. Riplinger, *J. Chem. Phys.*, 2020, **152**, 224108.
22. J. W. Furness, A. D. Kaplan, J. Ning, J. P. Perdew and J. Sun, *J. Phys. Chem. Lett.*, 2020, **11**, 8208-8215.
23. B. Benediktsson and R. Bjornsson, *J. Chem. Theory Comput.*, 2022, **18**, 1437-1457.
24. F. Neese, 2003, **24**, 1740-1747.
25. F. Weigend and R. Ahlrichs, *Phys. Chem. Chem. Phys.*, 2005, **7**, 3297-3305.
26. D. A. Pantazis and F. Neese, *Theor. Chem. Acc.*, 2012, **131**, 1292.
27. E. van Lenthe, E. J. Baerends and J. G. Snijders, *J. Chem. Phys.*, 1993, **99**, 4597-4610.

28. S. Grimme, J. Antony, S. Ehrlich and H. Krieg, *J. Chem. Phys.*, 2010, **132**, 154104.
29. D. A. Pantazis, X.-Y. Chen, C. R. Landis and F. Neese, *J. Chem. Theory Comput.*, 2008, **4**, 908-919.
30. E. Caldeweyher, C. Bannwarth and S. Grimme, *J. Chem. Phys.*, 2017, **147**, 034112.
31. S. Ehlert, U. Huniar, J. Ning, J. W. Furness, J. Sun, A. D. Kaplan, J. P. Perdew and J. G. Brandenburg, *J. Chem. Phys.*, 2021, **154**, 061101.
32. H. Li and J. H. Jensen, *Theor. Chem. Acc.*, 2002, **107**, 211-219.
33. Y. Ilina, C. Lorent, S. Katz, J.-H. Jeoung, S. Shima, M. Horch, I. Zebger and H. Dobbek, *Angew. Chem. Int. Ed.*, 2019, **58**, 18710-18714.
34. P. Rodríguez-Maciá, K. Pawlak, O. Rüdiger, E. J. Reijerse, W. Lubitz and J. A. Birrell, *J. Am. Chem. Soc.*, 2017, **139**, 15122-15134.
35. J. A. Birrell, V. Pelmenschikov, N. Mishra, H. Wang, Y. Yoda, K. Tamasaku, T. B. Rauchfuss, S. P. Cramer, W. Lubitz and S. DeBeer, *J. Am. Chem. Soc.*, 2020, **142**, 222-232.
36. M. Winkler, M. Senger, J. Duan, J. Esselborn, F. Wittkamp, E. Hofmann, U.-P. Apfel, S. T. Stripp and T. Happe, *Nature Commun.*, 2017, **8**, 16115.
37. S. P. Albracht, W. Roseboom and E. C. Hatchikian, *J. Biol. Inorg. Chem.*, 2006, **11**, 88-101.
38. P. Freire, F. Moreira Barboza, J. Alves de Lima Jr, F. Melo and J. Filho, 2017, in Raman Spectroscopy and Applications. InTech.
39. S. Yoshikawa, D. H. O'Keeffe and W. S. Caughey, *J. Biol. Chem.*, 1985, **260**, 3518-3528.
